# Evolutionary Processes Acting on Candidate *cis*-Regulatory Regions in Humans Inferred from Patterns of Polymorphism and Divergence

**DOI:** 10.1371/journal.pgen.1000592

**Published:** 2009-08-07

**Authors:** Dara G. Torgerson, Adam R. Boyko, Ryan D. Hernandez, Amit Indap, Xiaolan Hu, Thomas J. White, John J. Sninsky, Michele Cargill, Mark D. Adams, Carlos D. Bustamante, Andrew G. Clark

**Affiliations:** 1 Department of Molecular Biology and Genetics, Cornell University, Ithaca, New York, United States of America; 2 Department of Biological Statistics and Computational Biology, Cornell University, Ithaca, New York, United States of America; 3 Celera Diagnostics, Alameda, California, United States of America; University of California Davis, United States of America

## Abstract

Analysis of polymorphism and divergence in the non-coding portion of the human genome yields crucial information about factors driving the evolution of gene regulation. Candidate *cis*-regulatory regions spanning more than 15,000 genes in 15 African Americans and 20 European Americans were re-sequenced and aligned to the chimpanzee genome in order to identify potentially functional polymorphism and to characterize and quantify departures from neutral evolution. Distortions of the site frequency spectra suggest a general pattern of selective constraint on conserved non-coding sites in the flanking regions of genes (CNCs). Moreover, there is an excess of fixed differences that cannot be explained by a Gamma model of deleterious fitness effects, suggesting the presence of positive selection on CNCs. Extensions of the McDonald-Kreitman test identified candidate *cis*-regulatory regions with high probabilities of positive and negative selection near many known human genes, the biological characteristics of which exhibit genome-wide trends that differ from patterns observed in protein-coding regions. Notably, there is a higher probability of positive selection in candidate *cis*-regulatory regions near genes expressed in the fetal brain, suggesting that a larger portion of adaptive regulatory changes has occurred in genes expressed during brain development. Overall we find that natural selection has played an important role in the evolution of candidate *cis*-regulatory regions throughout hominid evolution.

## Author Summary

It has been suggested that changes in gene expression may have played a more important role in the evolution of modern humans than changes in protein-coding sequences. In order to identify signatures of natural selection on candidate *cis*-regulatory regions, we examined single nucleotide polymorphisms obtained from the complete re-sequencing of conserved non-coding sites (CNCs) in the flanking regions of over 15,000 genes in 35 humans. Patterns of allele frequencies in CNCs indicate the presence of both positive and negative selection acting on standing variation within these candidate *cis*-regulatory regions, particularly for the 5′ and 3′ UTRs of genes. Gene-specific tests comparing levels of polymorphism and divergence identify several genes with strong signatures of selection on candidate *cis*-regulatory regions and suggest that the biological characteristics of genes subject to selection are different between coding and candidate *cis*-regulatory regions with respect to gene expression and function. For example, we find stronger signatures of positive selection in candidate *cis*-regulatory regions near genes expressed in the fetal brain, which we do not observe in a concurrent analysis on protein-coding regions. Our results suggest that both positive and negative selection have acted on candidate *cis*-regulatory regions and that the evolution of non-coding DNA has played an important role throughout hominid evolution.

## Introduction

Over 30 years ago it was suggested that evolutionary changes at the level of gene regulation might have had a greater influence on human and chimpanzee phenotypic divergence than changes in proteins themselves [1]. This is exemplified by humans and chimpanzees being highly similar at the protein level yet manifesting considerable phenotypic differences. Until recently, the focus of evolutionary studies has been on changes occurring within the protein-coding regions of genes, while evidence from Drosophila [2],[3], rodents [4],[5], and primates [6]–[19] have all suggested that Darwinian selection can be an important driving force of evolutionary change within non-coding DNA. Microarray studies have identified numerous transcriptional differences between human and chimpanzee [20]–[25], and polymorphisms in *cis*-regulatory regions have been directly associated with differences in gene expression levels in humans [26],[27]. Therefore, single nucleotide polymorphisms (SNPs) in non-coding DNA can be associated with phenotypic differences, making them potential targets for natural selection.

A simple model of gene regulation involves the binding of transcription factors to several short non-coding sequences, which are generally found upstream of the transcribed regions of genes (forming a *cis*-regulatory region). Once a specific combination of transcription factors has bound to the DNA, the recruitment and assembly of the general transcriptional machinery will initiate gene transcription. However, gene regulation can also occur via additional processes, such as nucleosome positioning, distal enhancer elements, DNA methylation, and microRNA regulation. While a variety of computational methods have been developed to identify specific regulatory elements at the genomic scale, these methods can be prone to false positives due to the degeneracy and short size of DNA binding sites [28]. An alternative predictor of regulatory function at the genomic scale may be the evolutionary conservation between species [29],[30], although conservation scores can be prone to false negatives as not all functional sites are expected to be conserved [14],[31]. However, the study of conserved non-coding sites within regions potentially enriched for *cis*-acting elements does not *a priori* specify any particular mode of regulation, and may provide a broader glimpse of the evolutionary processes acting on candidate *cis*-regulatory regions.

We examined patterns of polymorphism and divergence in conserved non-coding sites (CNCs) in the flanking regions of 15,061 human genes, and the coding regions of 13,009 genes that were sequenced by Celera Genomics in 15 African Americans and 20 European Americans. We find evidence for selective constraint and adaptive evolution within candidate *cis*-regulatory regions, and find non-random patterns with respect to functional and transcriptional profiles of genes with higher probabilities of selection. Moreover, we find that patterns observed in candidate *cis*-regulatory regions are often distinct from those observed in protein-coding regions.

## Results/Discussion

**Figure 1:**
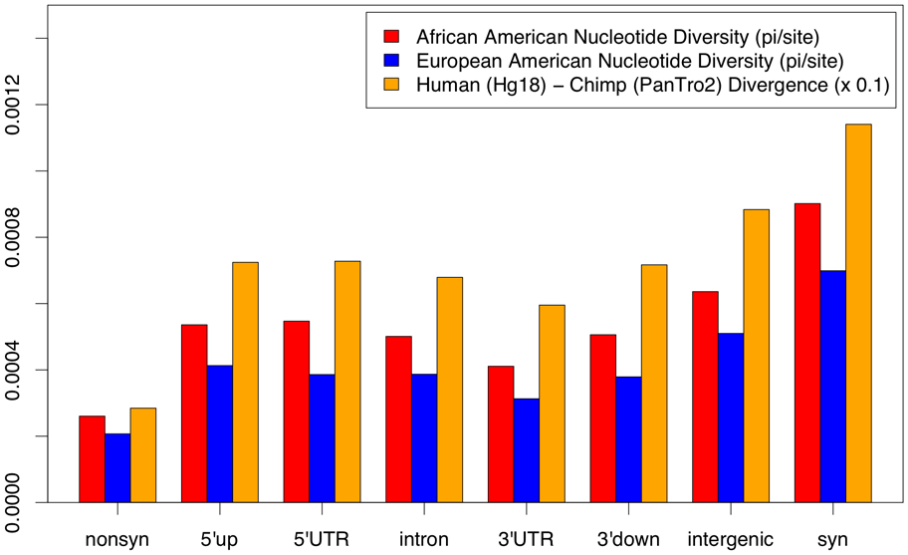
Polymorphism and divergence across pooled sites in African and European Americans.

Our non-coding dataset included 6.9 Mb of autosomal conserved non-coding sites (CNCs) resequenced in 15 African Americans (AAs), 20 European Americans (EAs), and aligned to a single chimpanzee (PanTro2), while our coding dataset included 17.5 Mb of autosomal sites resequenced across the same panel of individuals, see [32],[33]. Conserved noncoding sites were defined as strictly non-coding sites that fall within human and mouse conserved sequences (sequences with at least 70% identity and 100 nucleotides in length). We found at least one human polymorphism or fixed difference to the chimpanzee in CNCs flanking a total of 11,334 autosomal genes (75.4%), with 7,826 genes having at least one human polymorphism across the 35 individuals (52.9%). CNCs that are flanking genes tend to have lower divergence and polymorphism compared to those in intergenic regions, however all categories of CNCs exhibit lower polymorphism and divergence as compared to synonymous sites (Figure 1). In AAs, CNCs in 5′ upstream regions have a smaller ratio of divergence to polymorphism when compared to synonymous sites, consistent with the presence of selective constraint in the upstream regions of genes (*p* = 0.029, Table 1). However, the overall ratio of divergence to polymorphism is generally similar between pooled CNCs and synonymous sites (*p*>0.05, Table 1), and all categories of CNCs exhibit a higher ratio of divergence to polymorphism when compared to nonsynonymous sites.

**Table 1:** The total number of fixed and segregating sites in 15 African Americans (AA) and 20 European Americans (EA) as compared to the chimpanzee (PanTro2), along with the ratio of fixed/segregating (F/S) sites, the odds ratio (or neutrality index), and a chi-square test *p*-value for the comparison of F/S to synonymous sites.

Pop	Sites	Fixed	Segregating	F/S	Odds Ratio	p-value
**AA**	Synonymous	56124	19070	2.94	-	-
	Replacement	35195	15288	2.30	0.78	**<1×10^−6^** **
	Pooled CNCs	50249	17152	2.93	1.0	0.71
	5′ UTR	2355	854	2.76	0.94	0.11
	3′ UTR	2691	898	3.00	1.0	0.65
	5′ upstream	8117	2901	2.80	0.95	**0.029 ** *
	3′ downstream	3891	1299	3.00	1.0	0.59
	Intron	24919	8479	2.94	1.0	0.93
	Intergenic	14116	4665	3.03	1.0	0.14
**EA**	Synonymous	56690	13704	4.14	-	-
	Replacement	35554	12040	2.95	0.71	**<1×10^−6^** **
	Pooled CNCs	50649	12564	4.03	0.97	0.061
	5′ UTR	2366	570	4.15	1.0	0.94
	3′ UTR	2699	669	4.03	0.97	0.57
	5′ upstream	8182	2058	3.98	0.96	0.13
	3′ downstream	3915	922	4.25	1.0	0.49
	Intron	25121	6264	4.01	0.97	0.069
	Intergenic	14250	3429	4.16	1.0	0.83

*significant at the 5% level.

**significant at the 1% level.

**Figure 2:**
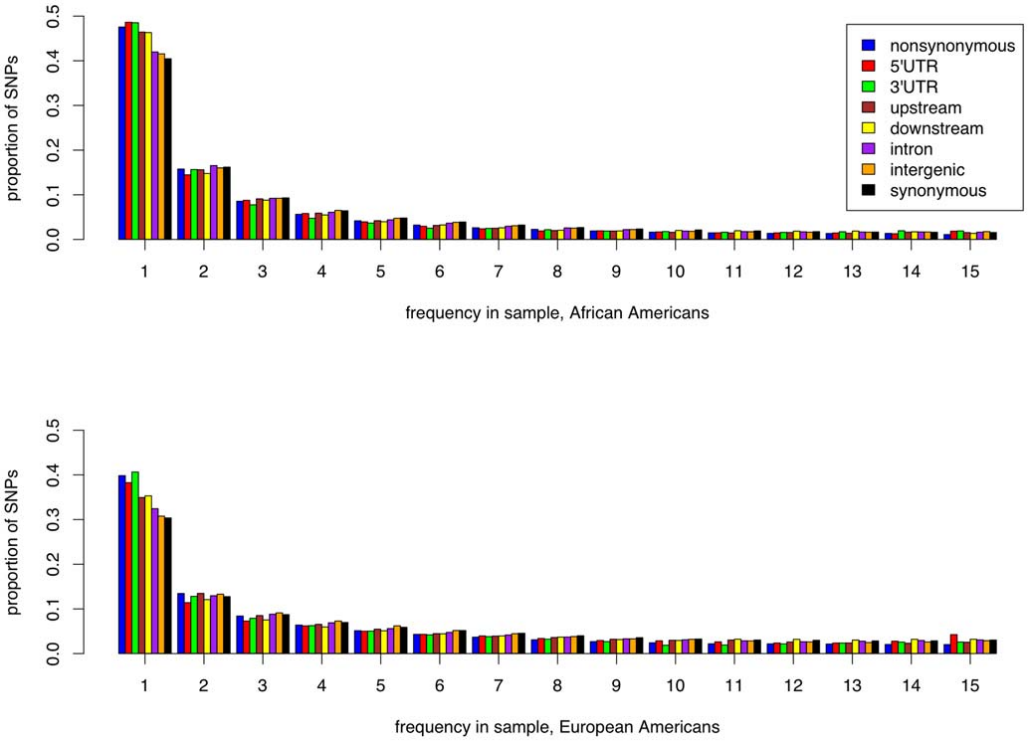
Allele frequency spectra for different classes of sites. Unfolded site frequency spectrum of nonsynonymous, synonymous, and conserved non-coding sites in 15 African Americans (above) and 20 European Americans (below); the data was re-sampled for 16 chromosomes to account for missing data. We used the chimpanzee to infer the ancestral state of each polymorphism, and corrected for ancestral misidentification using the method of Hernandez *et al.* [64].

The site frequency spectra of CNCs in both upstream and downstream regions of genes show a significant excess of lower frequency derived alleles (SNPs at a frequency of 1/16) as compared to both synonymous and intergenic sites (Figure 2 and Table 2), suggesting an excess of weakly deleterious alleles in CNCs in the flanking regions of genes. A study by Veyrieras *et al.* [34] has found that the majority of eQTLs lie either within or close to genes, suggesting that the excess of low frequency derived alleles observed in the flanking regions of genes may reflect the past action of negative selection on gene regulation. A shift in the distribution toward more rare alleles in CNCs is consistent with previous findings [8],[10],[13],[14], and confirmed by significantly smaller values of Tajima's *D* in CNCs as compared to synonymous sites (*D* = −0.52 vs. −0.45 in AAs [Mann-Whitney *U* test, *p*<10^−4^], *D* = −0.31 vs. −0.21 in EAs [Mann-Whitney *U* test, *p*<10^−6^]) (Figure S1). Therefore, patterns in the site frequency spectrum suggest that CNCs in the flanking regions of genes have been subject to selective constraint.

**Table 2:** Fisher's Exact tests comparing the proportion of low frequency derived alleles between different categories of CNCs compared to synonymous and intergenic sites.

Pop	CNCs	Synonymous OR (p-value)	Intergenic CNCs OR (p-value)
**AA**	pooled	1.06 (1.5×10^−3^)	1.03 (0.15)
	5′ upstream	1.15 (1.5×10^−4^)	1.12 (7.5×10^−3^)
	5′ UTR	1.20 (2.9×10^−3^)	1.17 (0.014)
	intron	1.04 (0.075)	1.01 (0.39)
	3′ UTR	1.20 (2.4×10^−3^)	1.17 (0.012)
	3′ downstream	1.15 (6.9×10^−3^)	1.12 (0.036)
	intergenic	1.03 (0.21)	-
**EA**	pooled	1.07 (7.2×10^−3^)	1.05 (0.11)
	5′ upstream	1.15 (2.3×10^−3^)	1.14 (0.015)
	5′ UTR	1.26 (4.0×10^−3^)	1.25 (9.4×10^−3^)
	intron	1.07 (0.022)	1.05 (0.13)
	3′ UTR	1.34 (1.4×10^−4^)	1.32 (6.3×10^−4^)
	3′ downstream	1.16 (0.018)	1.15 (0.040)
	intergenic	1.01 (0.39)	-

Low frequency derived alleles were defined as the number of SNPs at a frequency of 1/16 (i.e. frequency <10%) after resampling the data for 16 chromosomes to account for missing data in both African Americans (AA) and European Americans (EA). OR = odds ratio.

Estimates of the population scaled selection coefficient (γ = 2*N_e_s*) from the site frequency spectrum were calculated for different categories of CNCs using the program prfreq [32]. All point estimates of γ were negative for CNCs in the flanking regions of genes as compared to intergenic CNCs, which share the same ascertainment scheme and show no evidence for selection when compared to synonymous sites (Table 3). We find a significantly better fit for a single estimate of γ when compared to a neutral model for all categories of CNCs, suggesting the presence of selective constraint that cannot be explained by the ascertainment of CNCs alone. Furthermore, all but the 5′ upstream and 5′ UTR regions of genes show a significantly better fit to a model with a distribution of γ, implying there is variation in the deleterious effects of mutations in CNCs. We also observe an excess of fixed differences that cannot be explained by a Gamma model of deleterious fitness effects, suggesting the presence of positive selection within CNCs in the flanking regions of genes. We estimate that 4.67% of the human-chimp fixed differences in CNCs can be attributed to positive selection, with UTRs showing more adaptive evolution (5′: 14.3%, 3′: 23.3%) than upstream or downstream regions (5′: 12.2%, 3′: 12.0%). Intronic CNCs show less evidence for positive selection (2.22%), but because they make up the majority of sites, pooled CNCs in the flanking regions of genes show modest evidence for adaptive divergence relative to intergenic CNCs.

**Table 3:** Maximum likelihood estimates of γ under various models of fitness effects for mutations in CNCs in the flanking regions of genes as compared to intergenic sites.

Sites	MLE γ	ΔLL	MLE γ(α,β)	ΔLL	Expected Fixed	Observed Fixed	Excess Fixed
pooled	−0.897	131	(0.0415, 640)	66.4	48166.6	50414.5	4.67%
5′ upstream	−0.961	24.4	(0.172, 11.6)	4.1	7257.2	8143.4	12.2%
5′ UTR	−0.982	7.32	(0.184, 10.8)	1.2	2065.9	2360.6	14.3%
intronic	−1.16	116	(0.0219, 10∧6)*	92.3	24458.1	25001.7	2.22%
3′ UTR	−2.08	43.1	(0.117, 370)	22.9	2188.0	2698.5	23.3%
3′ downstream	−1.13	16.2	(0.061, 427)	10.4	3483.5	3902.9	12.1%
Intergenic	0.0315	0.04	-	-	-	-	-

The estimate of γ for intergenic sites is from a comparison to synonymous sites.

*asymptotes to β→∞.

### Gene-specific tests of selection

**Figure 3:**
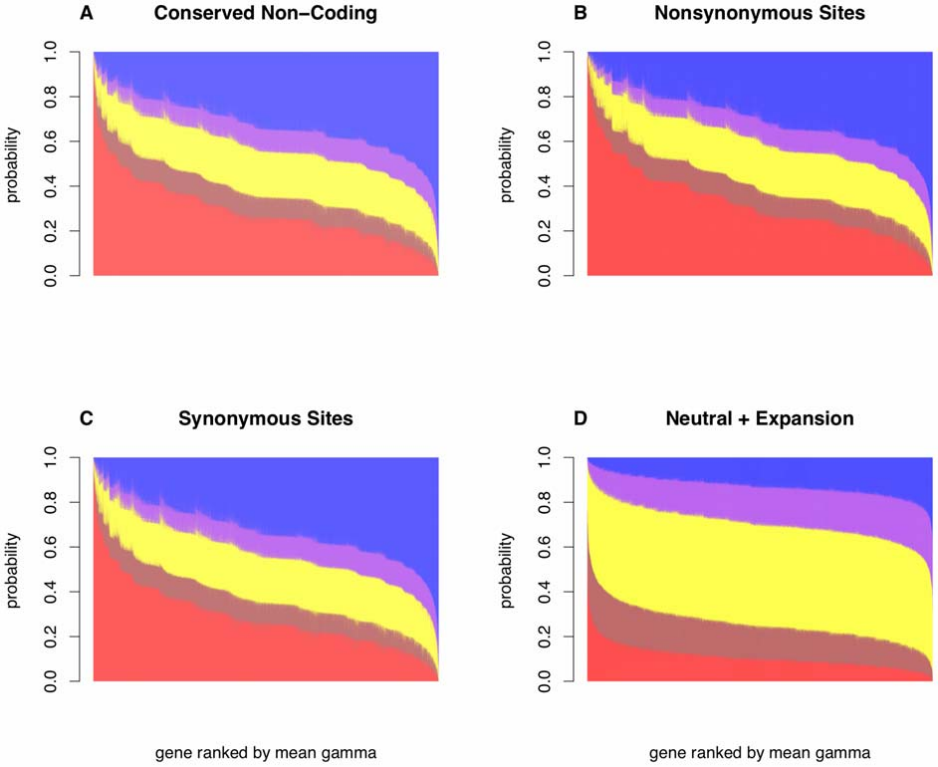
Gene-specific estimates of selection in mkprf. Distribution of the probability that γ falls within 5 categories: strong negative selection (red, γ<−1), weak negative selection (brown, −1<γ<−0.5), nearly neutral (yellow, −0.5<γ<0.5), weak positive selection (purple, 0.5<γ<1), and strong positive selection (blue, γ>1). Data shown is for African Americans from a concurrent analysis including (A) candidate *cis*-regulatory regions, (B) nonsynonymous sites, and (C) synonymous sites, and an independent analysis including (D) simulated neutral data under the inferred demographic model.

We defined a candidate *cis*-regulatory region as all CNCs within the introns, UTRs, or within 5 kb up- or downstream of the transcription start or stop site of a gene. CNCs were pooled in this way to increase the power to detect selection, and to capture signals of selection without regards to a specific mode of *cis*-regulation. However, we note there is a significant correlation between the probability of selection estimated from the 5′ upstream regions and the combined set of CNCs (Figure S2). To identify genes whose candidate *cis*-regulatory regions may be under selection, contingency tables were constructed containing the counts of polymorphic sites and fixed differences to the chimpanzee in the pooled flanking CNCs of a gene. The total number of synonymous polymorphisms and fixed differences in coding regions (without respect to human and mouse sequence conservation) were pooled to use as a neutral standard as in [35]; the use of pooled vs. local synonymous sites has little effect on our estimates of selection (Figure S3). In order to identify loci showing signatures of natural selection, we implemented the program mkprf [36] by conservatively setting no fixed variance on the prior distribution of the population scaled selection coefficient (γ = 2*N_e_s*), which shrinks the estimates of γ (Figure S4). For each gene we quantified the probability that the estimate of γ falls within five bins: γ<−1 (strong negative selection), −1<γ<−0.5 (weak negative selection), −0.5<γ<0.5 (nearly neutral), 0.5<γ<1 (weak positive selection), and γ>1 (strong positive selection) (Figure 3). In order to compare different modes of selection, the probability of negative selection was defined as Pr(γ<−0.5), and the probability of positive selection was defined as Pr(γ>0.5).

In order to control for population size changes that may affect our estimates of positive and negative selection, we incorporated demographic parameters when calculating the likelihood of our observed data in mkprf, the effect of which can be seen in Figure S5. A population expansion is expected to increase levels of polymorphism at neutral sites to varying extents due to differences in local mutation rates. In a set of neutral loci simulated under a model of population expansion, failing to correct for demography results in a neutral locus showing strong signatures of negative selection with credibility intervals on the mean estimate of γ below 0 (Table 4), and a slight inflation in the probability that γ<0 at higher values (Figure S6). Demographic models including a population expansion in African Americans and a single population bottleneck in European Americans were fitted to the complete set of autosomal synonymous SNPs using the program prfreq [32] and incorporated into mkprf. However, a single bottleneck model was found to be a poor fit to the European American sample, and even a complex, multi-bottleneck model could not account for the excess of high frequency derived alleles at synonymous sites [32]. Therefore, we focus the majority of our conclusions on the African American sample. A complete list of genes including McDonald-Kreitman tables and mkprf results are available in Table S1, Table S2, Table S3, Table S4, Table S5, Table S6, Table S7, and Table S8.

**Table 4:** The number of genes showing strong signatures of positive and negative selection in conserved non-coding sites (CNCs), synonymous, and nonsynonymous sites (Nonsyn) in African Americans (AA) and European Americans (EA), and for simulated data (Sim) under the inferred AA demographic model (see Methods).

Pop	Dataset	Total Genes (+/−)	Mean γ (con)	CI>0 (con)	CI<0 (con)
**AA**	CNCs	10,760	0.32 (0.09)	8 (7)	7 (12)
	Synonymous	11,746	0.31 (0.11)	5 (5)	4 (15)
	Nonsyn	9,645	−0.70 (−0.08)	4 (3)	88 (26)
	Nonsyn (cons)	4,889	−19.3	0	2,176
**EA**	CNCs	10,593	0.35 (0.07)	6 (6)	4 (14)
	Synonymous	11,637	0.46 (0.10)	4 (4)	11 (24)
	Nonsyn	9,539	−1.1 (−0.17)	2 (3)	111 (41)
**Sim**	neut	9,863 (0/0)	0.044	0	0
	neut (null demog)	9,863 (0/0)	0.0025	0	1
	neut (vs. syn+del)	9,863 (0/0)	0.26	0	0
	neut+wkdel	9,707 (0/699)	−0.17	0	8
	neut+del	9,707 (0/1,485)	−0.34	0	15
	neut+stdel+pos	9,707 (1,824/4,632)	−1.3	267	491
	neut+del+pos	9,707 (1,824/1,485)	0.97	203	26
	neut+wkdel+pos	9,707 (1,824/699)	1.2	199	12
	neut+pos	9,707 (1,824/0)	1.3	206	8

CNCs, synonymous, and nonsynonymous sites were analyzed both independently, and in a single concurrent run of mkprf (con), both with no fixed variance on the prior distribution of γ. Nonsynonymous sites were also analyzed by restricting to sites within human and mouse conserved regions (cons). Neutral loci simulated under the inferred AA demographic model were analyzed with a demographic correction, without a demographic correction (null demog), and assuming negative selection on synonymous sites (vs. syn+del). Total genes = genes with at least 1 fixed or polymorphic site, +/− = number of loci simulated under positive and negative selection respectively, CI = 95% credibility interval on the estimate of mean γ.

In order to evaluate the performance of mkprf, we performed Wright-Fisher forward simulations under the inferred demographic model in African Americans using the program SFS_CODE [37]. We find a significant correlation between simulated and estimated mean values of γ in datasets including various combinations of loci simulated under positive and negative selection (Figure S7). However, in a dataset including an equal number of loci simulated under positive and negative selection, the correlation is stronger for positively selected loci as compared to negatively selected loci, most likely reflecting a limited ability to distinguish between strong vs. weak negative selection. There is also a 10-fold increase in the number of genes returned with credibility intervals (CIs) on the mean estimate of γ>0 compared to CIs<0 (neut+del+pos, Table 4), as genes subject to strong enough negative selection often have reduced levels of polymorphism and divergence. Moreover, of the 11,000 simulated loci in the neut+del+pos dataset, 96% of loci in the positively selected class (γ>0) had at least 1 informative site, whereas only 94% of loci in the negatively selected class (γ<0) had at least 1 informative site making an estimate of γ even possible in mkprf. We note that across all datasets, only a limited number of the loci simulated under positive and negative selection have CIs on the mean estimate of γ above or below 0 (Table 4), suggesting there are likely additional loci subject to selection than identified by CIs alone.

An important consideration in gene specific tests for selection is unequal sequence coverage, as genes with a smaller number of resequenced sites tend to have fewer informative sites (fixed or polymorphic sites, Figure S8). It is unlikely that we have complete coverage of all *cis*-regulatory regions of a gene due to a focus on the 5′ upstream regions of genes, which is potentially problematic for our comparisons if genes with fewer informative sites were to provide less reliable estimates of γ. As discussed in the previous paragraph this may be particularly relevant for negatively selected loci, as genes subject to strong negative selection are expected to have fewer informative sites. Our simulations confirm that a higher percentage of negatively selected loci have fewer than 4 informative sites (neut+del+pos, 52%) as compared to either positively selected (16%) or neutral loci (28%). However, we find no significant difference in the distribution of γ when we compare loci with fewer or at least 4 informative sites when simulated under neutral, positive, and negative selection (Figure S9), suggesting that loci with fewer informative sites are unlikely to be problematic in our comparisons. The distributions of the number of resequenced sites at a locus in candidate *cis*-regulatory and protein-coding regions are shown in Figure S10.

### Selection on candidate *cis*-regulatory regions and gene expression profiles

We downloaded the Novartis Gene Expression Atlas 2 data from 72 normal human tissues in order to examine patterns of selection in candidate *cis*-regulatory regions with respect to gene expression signals [38]. Microarray expression profiles were available for 87% of genes in our tests for natural selection. Conflicting studies have shown that expression patterns in tissue-specific genes evolve more rapidly [39], or more slowly [40] as compared to broadly expressed genes in comparisons of humans and mice. More recently, experimentally defined *cis*-regulatory regions were found to exhibit stronger degrees of selective constraint in genes expressed in a smaller number of tissues [18]. However, we find little correlation between the probability of negative selection and the absolute number of tissues in which a gene is expressed (AA: Kendall's *tau* = −0.011, *p* = 0.12; EAs: *tau* = −0.0060, *p* = 0.40), nor with the index of tissue specificity [41], which includes additional information on the level of expression in each tissue (AA: Kendall's *tau* = 0.0086, *p* = 0.21; EAs: *tau* = 0.0024, *p* = 0.73). We also find no significant correlation between probabilities of selection and the mean and the maximum expression level of a gene across all tissues, suggesting that expression levels may not have a large impact on patterns of recent natural selection within candidate *cis*-regulatory regions. However, our simulations suggest that we likely have reduced power to identify significant correlations that are driven by negative rather than positive selection.

We then assigned genes to each tissue of expression in order to examine differences across tissues in evidence for selection on candidate *cis*-regulatory regions (Table S9, Table S10). Mann-Whitney *U* tests did not identify any tissues with a significantly higher probability of negative selection in either candidate *cis*-regulatory regions or nonsynonymous sites, suggesting that weak selective constraint may be a persistent factor affecting most human tissues, but likely reflects the limited power we have to detect negative vs. positive selection. On the other hand, we find that genes expressed in at least 3 tissues have a significantly higher probability of positive selection in candidate *cis*-regulatory regions (FDR<10%, Table 5), suggesting they may have an excess of positively selected loci. Notably, genes expressed in the fetal brain have a higher mean probability of positive selection as compared to genes expressed in other tissues, suggesting the importance of adaptive regulatory changes during brain development. Similarly, genes expressed in certain tissues of the adult brain, including the cerebellum peduncles and the medulla oblongata, also have a higher probability of positive selection in candidate *cis*-regulatory regions. Curiously, the medulla controls a variety of autonomic functions and is considered to be the most plesiomorphic structure of the brain [42], and might be expected to have a high level of conservation across species. However, the medulla also contains several motor nuclei important for facial expression, mastication, tongue movements, and controlling sound amplitudes that reach the inner ear, which are hypothesized to have played an adaptive role in the evolution of facial expression, feeding, and speech in humans [43].

**Table 5:** Tissues from the Novartis Gene Atlas 2 data with a higher mean probability of positive selection in candidate *cis*-regulatory regions (CNCs) and nonsynonymous sites (Nonsyn) in African Americans (AA) and European Americans (EA) (*p*<0.05, Mann-Whitney *U*-tests).

Sites	Tissue	AA *p*-value (*q*-value)	EA *p*-value (*q*-value)
**CNCs**	Fetal brain**	3.9×10^−4^ (0.015)	2.2×10^−3^ (0.15)
	Medulla oblongata**	4.1×10^−4^ (0.015)	0.021 (0.26)
	Cerebellum peduncles*	3.0×10^−3^ (0.073)	0.032 (0.26)
	Caudate nucleus	6.8×10^−3^ (0.12)	NT
	Pons	0.012 (0.17)	NT
	Testis seminiferous tubule	0.014 (0.17)	NT
	Cingulate cortex	0.017 (0.18)	0.036 (0.26)
	Amygdala	0.020 (0.18)	0.017 (0.26)
	Prefrontal cortex	0.028 (0.18)	NT
	Subthalamic nucleus	0.029 (0.18)	NT
	**Cerebellum**	0.030 (0.18)	NT
	Thalamus	0.032 (0.18)	NT
	Occipital lobe	0.036 (0.18)	NT
	**Whole brain**	0.039 (0.18)	0.030 (0.26)
	**CD4+ T cells**	0.041 (0.18)	5.9×10^−3^ (0.15)
	**CD56+ natural killer cells**	0.043 (0.18)	NT
	Spinal cord	0.043 (0.18)	NT
	Parietal lobe	0.046 (0.19)	0.043 (0.26)
	CD105+ endothelial	NT	6.4×10^−3^ (0.15)
	Thyroid	NT	0.027 (0.26)
	Testis germ cell	NT	0.041 (0.26)
**Nonsyn**	**CD4+ T cells**	0.012 (0.27)	NT
	**CD56+ natural killer cells**	0.012 (0.27)	NT
	CD71+ early erythroid	0.015 (0.27)	NT
	CD8+ T cells	0.021 (0.27)	NT
	Thymus	0.021 (0.27)	NT
	Lung	0.023 (0.27)	NT
	**Whole Brain**	0.034 (0.34)	NT
	CD34+ bone marrow	0.043 (0.34)	NT
	**Cerebellum**	0.048 (0.34)	NT

Tissues in bold are those that show a similar trend towards a higher probability of positive selection in both coding and candidate *cis*-regulatory regions. ** = FDR<5%, * = FDR<10%, NT = no trend with *p*-value>0.05.

Microarray studies have found that differences in brain expression patterns are more pronounced between humans and other primates as compared to other tissues [20], and that the majority of these differences are likely due to upregulation of brain-expressed genes in humans as compared to chimpanzees [21]. However, these findings have been controversial [22], and a more recent study has found that differences in expression patterns between humans and chimpanzees are less pronounced in the brain as compared to heart, kidney, liver and testis [24]. Regional expression in parts of the brain is generally conserved between human and the more distantly related mouse [44], raising the possibility that cognitive differences between species may be more likely to result from differential gene expression during development. While comparative microarray studies have generally focused on adult brain expression, our findings suggest that adaptive evolution might have had a larger impact on expression patterns in the fetal brain.

We applied the same methodology to examine differences in selection acting on coding regions with regards to gene expression. In contrast to what we observed for candidate *cis*-regulatory regions, we find no evidence for a higher probability of positive selection on nonsynonymous sites in genes expressed in the fetal brain in either AAs (Mann-Whitney *U*-test: *p* = 0.26) or EAs (*p* = 0.78). Our results are similar to a previous finding that positive selection in protein-coding regions is not elevated in genes expressed in the fetal brain [45]. In light of our results, it would seem that adaptive evolution of fetal brain development is influenced more strongly by changes at the regulatory level rather than at the protein-coding level. However, we note that genes expressed in the “whole brain” and cerebellum show a trend towards higher probabilities of positive selection within nonsynonymous sites in AAs (*p* = 0.03 and 0.048 respectively, FDRs = 34%), suggesting that adaptive changes in the human brain may be the cumulative result of positive selection on regulatory regions during early development, and possibly on protein-coding regions in the adult brain.

Previous studies have identified immune response and T cell-mediated immunity as processes that are enriched for genes showing signatures of positive selection in protein-coding regions [46],[47], highlight the importance of adaptive evolution in response to pathogens. We find that genes expressed in natural killer cells and T-cells both rank high among tissues with higher probabilities of positive selection in both coding and candidate *cis*-regulatory regions as compared to genes expressed in other tissues. Therefore, while genes expressed in the fetal brain show a different trend in coding and candidate *cis*-regulatory regions, positive selection in genes expressed in various immune cells may have occurred at both the coding and regulatory level.

### Selection on candidate *cis*-regulatory regions and functional categories

Evolutionary patterns within candidate *cis*-regulatory regions can also provide insight into the relative importance of functional categories in the evolution of modern humans. We generated a custom GOslim set containing 129 terms from the Gene Ontology database [48], and performed Mann-Whitney *U*-tests to identify functional categories that have higher than expected probabilities of selection within candidate *cis*-regulatory regions (Table S11, Table S12). In AAs, functional categories with a higher probability of positive selection in candidate *cis*-regulatory regions include regulation of cellular process (GO:0050794, *p* = 5.6×10^−4^, FDR = 4%), protein modification (GO:0006464, *p* = 4.8×10^−3^, FDR = 9%), and cell cycle (GO:0007049, *p* = 3.7×10^−3^, FDR = 9%) (Table 6). In EAs, the most significant terms include calcium ion binding (GO:0005509, *p* = 4.9×10^−3^, FDR = 18%), organelle organization and biogenesis (GO:0006996, *p* = 5.4×10^−3^, FDR = 22%), cell cycle (GO:0007049, *p* = 7.9×10^−3^, FDR = 22%), and behavior (GO:0007610, *p* = 9.3×10^−3^, FDR = 22%). Functional categories with a higher probability of negative selection in candidate *cis*-regulatory regions in AAs are generally less significant than positive selection, but include cytosol (GO:0005829, *p* = 8.6×10^−3^, FDR = 16%), ribosome (GO:0005840, *p* = 0.02, FDR = 16%), extracellular region (GO:0005576, *p* = 0.03, FDR = 16%), and carrier activity (GO:0005386, *p* = 5.9×10^−3^, FDR = 22%), and in EAs include proteinaceous extracellular matrix (GO:0005578, *p* = 0.01, FDR = 19%), and extracellular space (GO:0005615, *p* = 0.03, FDR = 19%) (Table 6).

**Table 6:** Gene Ontology terms with higher mean probabilities of positive and negative selection in candidate *cis*-regulatory regions (CNCs) and nonsynonymous sites (Nonsyn) in African Americans (AA) and European Americans (EA) (FDR<25%, Mann-Whitney *U* test). ** = FDR<5%, * = FDR<10%.

Pop	Sites	Negative Selection	Positive Selection
**AA**	**CNCs**	Carrier activity	Regulation of cellular process**
		Cytosol	Protein modification*
		Ribosome	Cell cycle*
		Extracellular region	Cell division*
		DNA binding	Negative regulation of biological process
		Nucleotide binding	Enzyme regulator activity
			Ligase activity
			Calcium ion binding
	**Nonsyn**	Actin binding	Transcription*
		Protein complex	Transcription factor activity
		Channel or pore class transporter activity	Receptor activity
		Oxidoreductase activity	
		Structural molecule activity	
**EA**	**CNCs**	Proteinaceous extracellular matrix	Calcium ion binding
		Extracellular space	Organelle organization and biogenesis
			Cell cycle
			Behavior
	**Nonsyn**	Calcium ion binding*	Immune system process*
		Catabolic process	Response to stress*
		Nervous system development	Transcription*
		Positive regulation of transcription, DNA-dependent	Defense response*
			Regulation of transcription*
			Response to stimulus*
			Response to external stimulus*
			Transcription factor activity
			Carbohydrate binding
			Phosphorylation

We tested the same group of GO terms in our parallel tests for selection on nonsynonymous sites within coding regions, and find that different categories are identified as having higher probabilities of selection in nonsynonymous sites as compared to candidate *cis*-regulatory regions (Table 6). Terms showing significantly higher probabilities of positive and negative selection in nonsynonymous sites correspond to previously published results involving the same dataset with AAs and EAs pooled [35]. For example, we find that “transcription” has significantly higher probabilities of positive selection in nonsynonymous sites (GO:0006350; AA: *p* = 1.1×10^−3^, FDR = 8%; EA: *p* = 2.5×10^−3^, FDR = 6%), and that “actin binding” has higher probabilities of negative selection in nonsynonymous sites (GO:0003779: *p* = 8.0×10^−3^, FDR = 25%). Transcription factors have frequently been reported as having a high degree of positive selection in protein-coding regions in general [35],[45],[49],[50], however this is not a trend that we observe in candidate *cis*-regulatory regions. Interestingly, in EAs we find that “calcium ion binding” has a significantly higher probability of negative selection at nonsynonymous sites (GO:0005509, *p* = 1.5×10^−3^, FDR = 6%), but within candidate *cis*-regulatory regions it shows a higher probability of positive selection (*p* = 4.9×10^−3^, FDR = 18%). Therefore, evolutionary patterns in candidate *cis*-regulatory regions tend to exhibit different functional patterns than those found in protein coding regions of genes.

### Noteworthy genes with evidence for selection on candidate *cis*-regulatory regions

We identified several genes that are likely to have undergone adaptive regulatory changes during human evolution (95% credibility interval on mean γ above 0). *NUDT16* shows the strongest evidence for positive selection in candidate *cis*-regulatory regions in both AAs (Pr[γ>0.5] = 98.5%) and EAs (Pr[γ>0.5] = 97.4%). *NUDT16* is a negative regulator of ribosome biogenesis, and may be involved in RNA decay in the nucleus. *ITPR1* shows the second strongest evidence for positive selection in EAs (Pr[γ>0.5] = 97.3%), however we find much weaker evidence for positive selection in AAs due to the presence of SNPs that are exclusive to AAs (Pr[γ>0.5] = 70.7%). *ITPR1* is essential for brain function, and is translated in response to synaptic activity in order to modulate calcium release from the endoplasmic reticulum. However *ITPR1* also modulates calcium entry in the plasma membrane of B-cells, suggesting it may also have an important role in immunity. Functional studies have shown that the 3′ UTR of *ITPR1* is required for dendritic localization in the mouse, however the majority of human-chimp fixed differences fall within conserved intronic sites rather than in the 3′ UTR region of this gene, suggesting that the target of selection is more likely to be an intronic *cis*-acting element (or elements). Within nonsynonymous sites, *ITPR1* demonstrates little evidence for either negative or positive selection in both AAs and EAs, suggesting that positive selection on *ITPR1* likely involved adaptive substitutions within candidate *cis*-regulatory regions rather than within protein-coding regions.

Additional genes with strong evidence for positive selection in candidate *cis*-regulatory regions in both populations include *CACNA2D3*, a calcium channel subunit protein ubiquitously expressed in fetal tissues; *OR2L13*, an olfactory receptor; and *RNF167*, a gene involved in protein degradation.

We also identified several genes that are likely to have experienced negative selection on candidate *cis*-regulatory regions (95% credibility interval on mean γ below 0). The gene with the highest probability of negative selection in candidate *cis*-regulatory regions in AAs is *FREM1* (Pr[γ<−0.5] = 98.4%), a gene involved in the development of a number of epidermal structures in the mouse [51], which also shows signatures of negative selection in EAs (Pr[γ<−0.5] = 92.9%, but CI includes 0). *FREM1* is highly expressed in the dermis during mouse embryonic development, and truncation of the FREM1 protein results in blebbing (blistering) diseases that are similar to phenotypes observed in Fraser syndrome, and dystrophic epidermolysis bullosa in humans. The gene with the highest probability of negative selection in candidate *cis*-regulatory regions in EAs in *KRT40* (Pr[γ<−0.5] = 99.9%), a hair keratin protein that shows a similarly high probability of negative selection in AAs (Pr[γ<−0.5] = 97.6%). However, positive values of Tajima's *D* in both EAs (*D* = 1.80) and AAs (*D* = 1.32) indicate that polymorphisms at *KRT40* tend toward intermediate frequencies, suggesting that *KRT40* may potentially be subject to balancing rather than negative selection.

Other genes with strong evidence for negative selection in candidate *cis*-regulatory regions include *SGCZ*, a gene that may be important in the pathogenesis of muscular dystrophy and cardiomypathy; *KIF19*, a gene involved in intracellular transport and a member of a superfamily of proteins important for brain functioning; *DOCK1*, a gene thought to play a role in regulating phagocytosis during apoptosis; and *TNNI3K*, a cardiac-specific protein kinase. We also identify *C14orf119* and *C20orf117* as having strong evidence of negative selection in candidate *cis*-regulatory regions, however these genes have not been fully characterized.

### Selection inferred at disease-associated genes

In order to examine patterns of natural selection on candidate *cis*-regulatory regions with respect to human disease, we identified 666 Mendelian disease genes using a hand-curated list of genes from the Online Mendelian Inheritance in Man database (OMIM) [52], and 1,072 complex disease genes using the Genetic Association Database (GAD) [53] that were included in our scans for selection. We find that disease genes have a higher mean probability of negative selection within candidate *cis*-regulatory regions as compared to non-disease genes, however this trend is only suggestive in EAs, the population where the majority of diseases have likely been characterized (Mann-Whitney *U*-test; OMIM: *p* = 0.23 in AAs, *p* = 0.011 in EAs; GAD: *p* = 0.29 in AAs, *p* = 0.06 in EAs). A link between negative selection and human disease has also been observed in protein-coding regions of the genome [35], however genetic diseases can also be regulatory in nature [54].

There are several examples of disease-associated genes showing evidence for negative selection in candidate *cis*-regulatory regions in EAs. For example, *LDB3*, is a gene expressed in skeletal and cardiac muscle for which several nonsynonymous mutations have been associated with myofibrillar myopathy (OMIM:609452) and cardiomyopathy (OMIM:601493). Another gene, *PLCE1* shows evidence for negative selection in both coding and candidate *cis*-regulatory regions, and homozygous mutations within the coding regions have been associated with type 3 nephrotic syndrome (OMIM:610725). Both *LDB3* and *PLCE1* show only moderate signatures of negative selection at candidate *cis*-regulatory regions in AAs.

On the other hand, there are several examples of disease genes that show strong evidence of positive selection in candidate *cis*-regulatory regions. For example, *CACNA2D3* displays a pattern where loss of heterozygosity in intronic sequences is associated with renal cell carcinoma; *CENTG3* variants confer protection against the pathogenesis of polyglutamine disease in the brain; and *ALG3* variants are associated with congenital disorder of glycosylation type Id, a metabolic disease.

### Contrasts of selection on protein-coding and candidate *cis*-regulatory regions

Direct comparisons between different classes of sites in mkprf may be confounded by differences in power to infer selection when there are varying degrees of selection on background loci in the dataset, particularly when there is no fixed variance on the prior distribution of γ. Our simulations show that as the proportion of negatively selected loci is varied, so does the number of genes showing strong signatures of positive selection due to a broader exploration of the parameter space (CI>0, Table 4), despite the actual number of positively selected loci remaining constant. For example, by increasing the number of negatively selected loci from 0 (neut+pos) to 699 (neut+wkdel+pos), to 1,485 (neut+del+pos), while keeping the number of positively selected loci constant (1,824 loci), the number of genes with CIs>0 changes from 206 to 199, to 203 (Table 4). If we increase the strength and the number of negatively selected loci to 4,632 loci (neut+stdel+pos), we then identify 267 loci with CIs>0 (Table 4). More importantly, the distributions of γ for positively selected and neutral loci show visible differences when the number of negatively selected loci is varied, which may confound direct comparisons when different classes of sites are analyzed independently (Figure S11). In order to control for varying levels of selection on background loci, we ran a combined analysis with nonsynonymous, synonymous, and candidate *cis*-regulatory regions in a single run of mkprf. The effect of analyzing different classes of sites in a concurrent vs. independent analysis is a shift in the distribution of γ towards smaller values for both candidate *cis*-regulatory regions and synonymous sites, and a shift in the distribution of γ towards larger values for nonsynonymous sites, causing the distributions of γ to be more similar, and more closely centered around 0 for different classes of sites (Figure S12).

**Figure 4:**
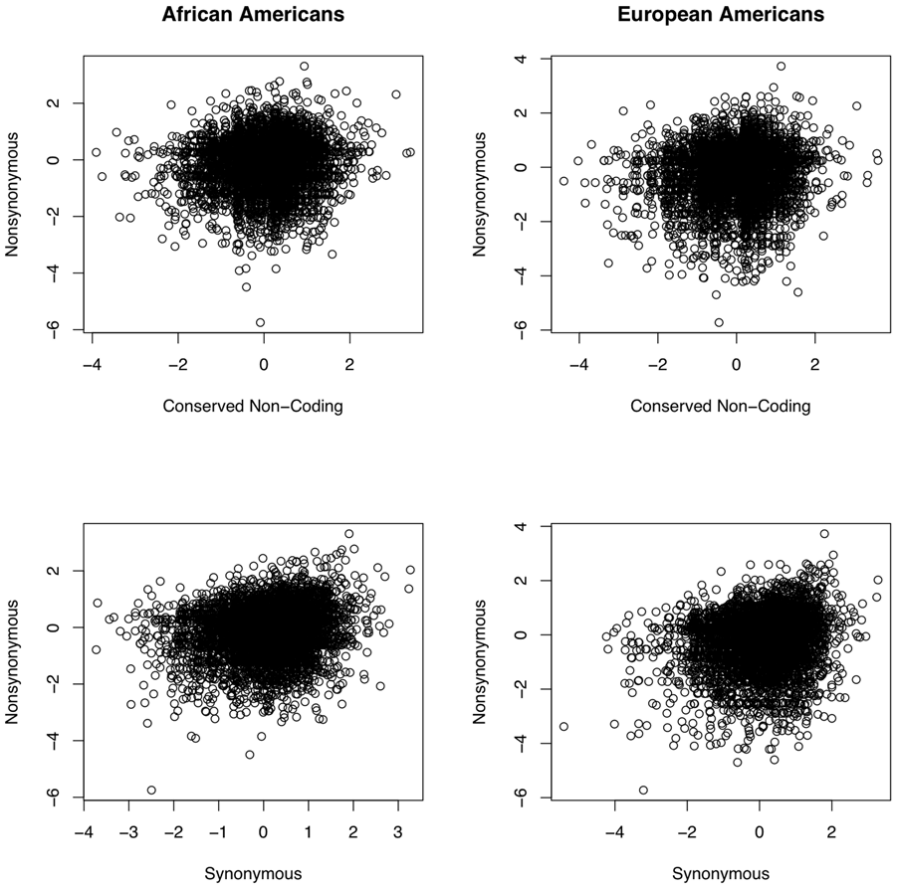
Correlation in estimates of γ at different classes of sites within a gene. There is a significant, yet weak positive correlation between estimates of γ in candidate *cis*-regulatory regions and nonsynonymous sites in both African Americans (top left) (Kendall's *tau* = 0.055, *p* = 1.8×10^−11^), and European Americans (top right) (*tau* = 0.043, *p* = 2.9×10^−7^). There is a slightly stronger correlation between synonymous and nonsynonymous sites in both African Americans (bottom left) (*tau* = 0.096, *p*<10^−16^), and European Americans (bottom right) (*tau* = 0.087, *p*<10^−16^). Candidate *cis*-regulatory, synonymous, and nonsynonymous sites were run in a single, concurrent run of mkprf.

First we looked for a correlation between selection acting on coding and candidate *cis*-regulatory regions, and observe what appears to be a complex relationship (Figure 4). In AAs we find a weak yet significant rank correlation between nonsynonymous and non-coding regions for both the probability of positive (Kendall's *tau* = 0.055, *p* = 1.6×10^−11^) and negative selection (*tau* = 0.056, *p* = 9.6×10^−12^), and between synonymous and non-coding regions (positive selection: *tau* = 0.060, *p* = 2.1×10^−13^; negative selection: *tau* = 0.060, *p* = 4.1×10^−13^). The correlation between synonymous and nonsynonymous sites appears to be slightly stronger (positive selection: *tau* = 0.098, *p*<10^−16^; negative selection: *tau* = 0.096, *p*<10^−16^), as expected due to increased linkage disequilibrium from a closer proximity between sites. Results from the EA sample are similar. However, given the small values of *tau*, identifying genes with evidence for selection in coding regions may be a poor predictor of whether a gene will show evidence for selection in the same direction as candidate *cis*-regulatory regions. A recent study has found that genes with patterns of expression consistent with any direction of selection (either positive or negative) exhibit reduced rates of protein evolution on nonsynonymous sites [25], which is consistent with only a weak correlation in the probability of selection between sites. The lack of a strong correlation in the mode of selection also suggests that the effect of linkage between candidate *cis*-regulatory and protein coding regions may be small, increasing the probability that we are detecting signatures of selection that are specific to non-coding regions.

**Figure 5:**
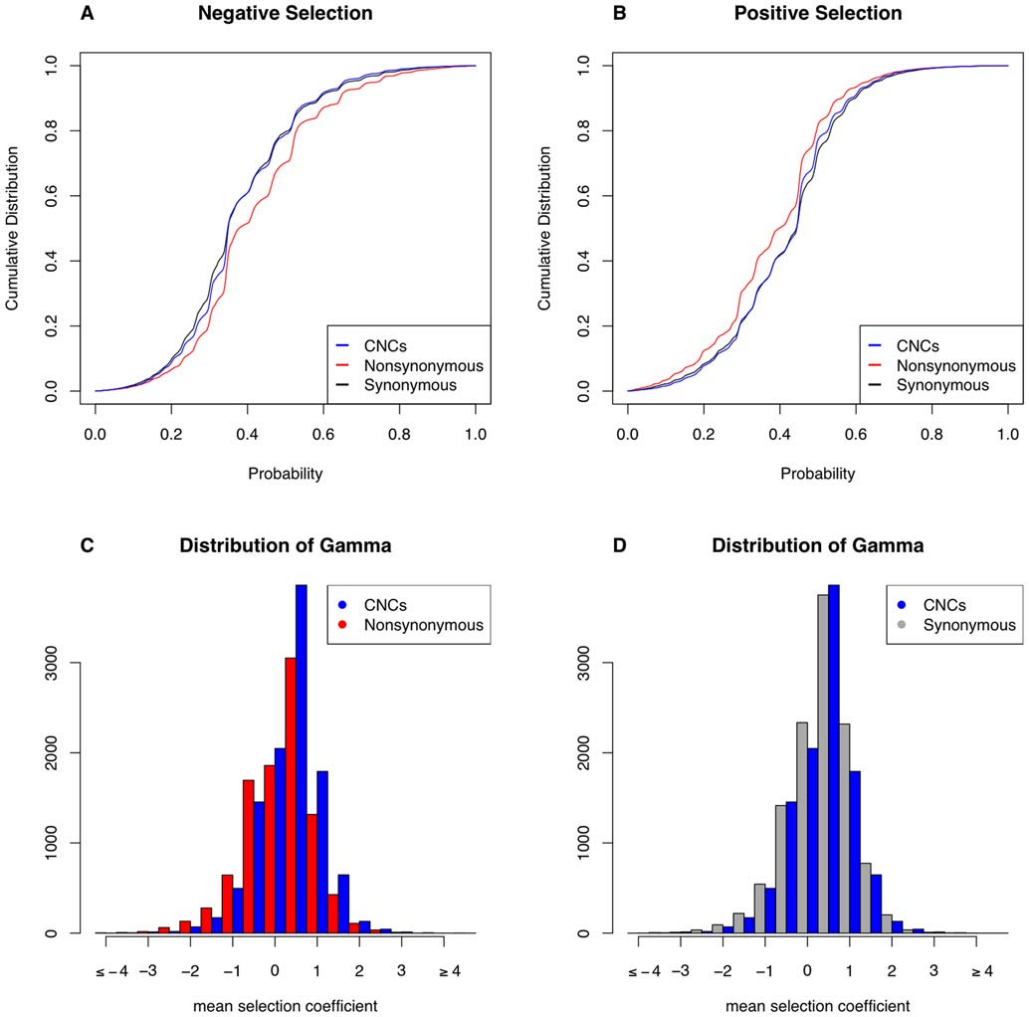
A comparison of signatures of selection between different classes of sites. Cumulative distributions of (A) the probability of negative selection [Pr(γ)<−0.5] and (B) the probability of positive selection [Pr(γ)>0.5] across different classes of sites, and the distribution of γ in (C) candidate *cis*-regulatory and nonsynonymous sites, and (D) candidate *cis*-regulatory and synonymous sites. Data shown is for African Americans from a single, concurrent analysis in mkprf including all classes of sites.

We next compared the overall distributions of the probability of selection at different classes of sites (Figure 5). Nonsynonymous sites have a higher mean probability of negative selection as compared to both candidate *cis*-regulatory regions and synonymous sites (Mann-Whitney *U*-tests, *p*-values<10^−16^), and candidate *cis*-regulatory regions have a significantly higher mean probability of negative selection as compared to synonymous sites (*p* = 8.7×10^−4^). We find that candidate *cis*-regulatory regions exhibit a significantly higher mean probability of positive selection as compared to nonsynonymous sites (*p*<10^−16^), however this is also true for synonymous sites (*p*<10^−16^), and synonymous sites have a marginally higher mean probability of positive selection as compared to candidate *cis*-regulatory regions (*p* = 0.014). Although synonymous sites may not evolve under strict neutrality, they may better represent the distribution expected under neutrality while taking into consideration local effects of linkage, mutation rates, and variability in effective population sizes across the genome. Therefore, differences in the distributions of the probability of positive selection between nonsynonymous and candidate *cis*-regulatory regions may be driven by more neutral, rather than more adaptive evolution in candidate *cis*-regulatory regions.

However, it is possible we have limited power to identify differences in the degree of positive selection in protein-coding and candidate *cis*-regulatory regions, as the extent of positive selection within candidate *cis*-regulatory regions may be an underestimate due to their ascertainment. By restricting our analyses to human-mouse conserved sequences, it may have biased our dataset toward lower ratios of divergence/polymorphism based on neutral coalescent simulations (Text S1, Table S13, Table S14, Table S15, Figure S13, and Figure S14). For example, if we restrict our analysis of nonsynonymous sites to those within human-mouse conserved regions (*i.e.* use the same ascertainment as non-coding sites), all genes show strong evidence for strong selective constraint and have 95% credibility intervals below 0 on the mean estimate of γ (Table 4). Moreover, it is likely that only a small proportion of sites within candidate *cis*-regulatory regions are truly functional, as regulatory elements are often small. Therefore, the relative contribution of adaptive evolution at the level of gene regulation vs. changes in the actual protein remains an open question.

An important consideration is whether selection on synonymous sites has affected our estimates of selection. While we observe no discernable relationship between the probability of positive or negative selection with GC content (Figure S15), negative selection may indeed affect a certain proportion of synonymous mutations [55]. Wright-Fisher simulations show that if purifying selection is acting on synonymous sites, we should expect a shift in the distribution of γ from a mean of 0.044 to 0.26 for a set of neutral loci (Table 4). On the other hand, selection on closely linked nonsynonymous sites may cause a reduction in polymorphism at synonymous sites, and could explain the signatures of positive selection we observe on synonymous sites (Table 4). Nevertheless, pooled synonymous sites provide a good fit to a neutral demographic model of expansion in the African American sample [32], and were used as a neutral standard in the same way for all classes of sites. Therefore, any bias is expected to have a similar affect on candidate *cis*-regulatory, synonymous, and nonsynonymous sites.

### Conclusion

Our analysis of human polymorphism and divergence in conserved non-coding sites suggests that the evolution of candidate *cis*-regulatory regions is often driven by both positive and negative selection. Our findings reinforce the idea that the non-coding portion of our genome has an important functional and evolutionary role, and suggest that patterns of natural selection in non-coding DNA are often distinct from that of protein-coding regions. Many of the adaptive changes in candidate *cis*-regulatory regions might have occurred near genes expressed in the fetal brain, supporting the hypothesis that the evolution of the developing brain may be largely attributable to changes in gene regulation. Our results add to the increasing evidence that non-coding DNA is not all selectively neutral, and that selection on candidate *cis*-regulatory regions has played an important role throughout hominid evolution.

## Methods

### Sequencing and bioinformatics

Sequencing and SNP detection was performed at Celera Genomics in 19 African Americans, 20 European Americans, and one chimpanzee as previously described [35]. Primers were designed to amplify the protein-coding exons of 23,363 genes according to Celera's human genome version R26k, which concurrently amplified non-coding flanking sequences around each exon. Primers were also designed to target human and mouse conserved sequences (HMCS) within 5 kb upstream of the first start codon (for a total of 9,459 genes). HMCS were defined as sequences >100 bp and >70% identity between human and mouse, however the majority were between 250–500 bp in length.

The bioinformatic pipeline is depicted in Figure S16. We downloaded over 525,000 human and mouse conserved sequences (HMCS) from the UCSC genome browser [56], of which 87,100 were aligned to the trace sequences using BLAST [57]. Sequences were oriented to hg17 using BLAT [58], of which 85,641 mapped to a unique position on the human genome. The positions of all but five HMCS were updated to hg18 using liftOver [59] with a ratio of remapped bases set to 1. A total of 83,379 HMCS were oriented to the public chimpanzee genome (PanTro2) using syntenic alignments from the UCSC genome browser. HMCS were annotated to genes according to Refseq 19 with respect to sites 5 kb upstream of the transcription start site, 5 kb downstream of the transcription stop site, 5′ UTR, 3′ UTR, intron, and coding. Sites beyond 5 kb upstream or downstream from any known transcript were annotated as intergenic. All sites within HMCS that overlapped with any known coding exons were masked, leaving strictly conserved non-coding sites (CNCs). Our resulting dataset included CNCs flanking a total of 15,061 autosomal genes. For comparisons to synonymous and nonsynonymous sites, we used the Celera Genomics exon resequence data from the same set of 35 people without regard to human and mouse conservation [35]. In the case of alternatively transcribed genes, we counted the total number of fixed and polymorphic sites in nonsynonymous sites as the union of all known transcripts. An analysis of admixture revealed that 4 out of the 19 African American individuals had high levels of European ancestry [33], so these individuals were excluded from our study.

### Statistical analyses

In order to estimate the distribution of fitness effects on conserved non-coding sites (CNCs) in the flanking regions of genes for African Americans, we calculated the likelihood that the observed site frequency spectrum fits a neutral model, a model with a single estimate of γ, and model with a Gamma distribution of γ using the program prfreq [32]. Estimates of γ for intergenic CNCs were calculated relative to synonymous sites, and yielded no evidence for natural selection on intergenic CNCs. Estimates of γ for CNCs in the flanking regions of genes were then calculated relative to intergenic CNCs rather than synonymous sites in order to control for the effect of ascertainment based on conservation. Demographic parameters were simultaneously inferred from intergenic CNCs, which produced a similar model to that inferred from synonymous sites.

In order to identify candidate *cis*-regulatory regions subject to positive and negative selection we implemented the program mkprf [36],[60], which estimates the posterior distribution of the population scaled selection coefficient (γ = 2N_e_s) for individual loci. For a neutral comparison we pooled the number of fixed and segregating synonymous sites from the exon resequencing data, similar to the approach taken in Bustamante *et al.* [35]. We updated the mkprf framework to incorporate the effect of non-stationary demography using the Poisson Random Field approach [61], and used the maximum likelihood demographic parameters from Boyko *et al.* [32] that were estimated from the frequency information at synonymous coding sites. In this model, the African population exhibited a 3-fold expansion approximately 6,800 generations ago whereas the European population exhibited a bottleneck followed by a more recent expansion. We implemented mkprf by conservatively setting no fixed variance on the prior distribution of γ, as the number of genes with credibility intervals (CIs) above or below 0 has been shown to be positively correlated to the variance set on the prior distribution of γ [62]. For example, by not fixing the variance we identify 8 genes with CIs>0 and 7 genes with CIs<0 in our analysis of CNCs, whereas by fixing the variance to 8 we identify 11 genes with CIs>0 and 60 genes with CIs<0.

### Simulations

We performed Wright-Fisher forward simulations under the inferred demographic model for AAs using the program SFS_CODE [37] (Table 4). For each scenario a total of 11,000 loci were simulated using the length distribution of resequenced sites in candidate *cis*-regulatory regions (or synonymous sites), the observed mutation rate from pooled synonymous sites (θ = 5.91×10^−4^), partial linkage between sites (rho = θ), and a splitting from the chimpanzee ancestor 20*2*N* generations ago to match the observed fixed/segregating ratio in pooled synonymous sites (*N* = 7,778, the estimated ancestral population size estimated from synonymous sites [32]). Two sets of loci were simulated under a neutral demographic model (one for candidate *cis*-regulatory regions, one for synonymous sites), and three sets of loci were simulated under different selective regimes (one for candidate *cis*-regulatory regions, one for nonsynonymous, and one for synonymous sites). For candidate *cis*-regulatory regions a distribution of selective effects were drawn from a mixture of normal distributions assuming that most loci were under weak selection or nearly neutral (*N* = 10,500, mean = 0, s.d. = 0.5), but with some loci having more extreme selection coefficients (*N* = 500, mean = 0, s.d. = 5). This distribution assumed an equal number of genes exposed to positive and negative selection, and allowed us to evaluate our method under a general condition (neut+del+pos, Table 4). Loci with no informative sites were discarded, leaving a total of 9,863 loci in the neutral demographic set, and 9,707 loci in the neutral demographic+selection set that had at least 1 fixed or polymorphic site.

In order to evaluate our method under different conditions, we randomly substituted half of the negatively selected genes for neutral loci (neut+wkdel+pos), half of the negatively selected and all of the positively selected genes for neutral loci (neut+wkdel), all of the negatively selected loci for neutral loci (neut+pos), and all of the positively selected loci for neutral loci (neut+del). For nonsynonymous sites a distribution of selective effects was drawn from an exponential distribution with rate parameter 0.2, and truncated to the nearest integer. We then substituted all of the negatively selected loci that were simulated for nonsynonymous sites for an equivalent number of neutral loci in the neut+pos dataset (neut+stdel+pos). For synonymous sites a distribution of selective effects was drawn from an exponential distribution with rate parameter 0.8, reflected across the *y*-axis to be negative, and truncated to the nearest integer.

### Correlations with gene attributes

All correlations with gene attributes were run using the continuous distribution of the probability of positive and negative selection (Pr[γ>0.5] and Pr[γ<−0.5]). A Kendall's *tau* rank correlation coefficient was calculated in order to test for correlations between the probability of positive or negative selection and continuous biological variables. Mann-Whitney *U*-tests were used to test for differences in the mean probability of selection with discrete data, and multiple testing was considered using the false discovery rate [63] in the R statistical package using q.value. We created a custom GOslim including 129 terms, and annotated genes to the most terminal child to look for functional categories with higher probabilities of natural selection. In order to examine patterns of selection with regards to the transcriptional profiles of genes, we downloaded the Novartis Gene Expression Atlas 2 data [38] from 72 normal human tissues from the UCSC genome browser [56]. A gene was considered to be expressed in a tissue if the signal was >350, and in the case of genes with multiple transcripts the average expression level for each tissue was calculated. The index of tissue specificity (τ) was estimated for each gene according to Yanai *et al.* [41].

## Supporting Information

Figure S1 Estimates of Tajima's D for different categories of sites; syn = synonymous sites, CNCs = conserved non-coding sites, repl = nonsynonymous sites. Notches represent the 95% confidence interval for the difference in two medians.
(6.22 MB TIF)

Figure S2 Comparison of the probability of negative selection (above) and the probability of positive selection (below) when only CNCs in the 5′ upstream regions of genes are considered, as compared to when all CNCs are pooled for African Americans in mkprf.
(4.11 MB TIF)

Figure S3 The effect of using pooled vs local synonymous sites on estimates of the probability of negative selection (above) and the probability of positive selection (below) for candidate *cis*-regulatory regions in the African American sample. The effect of pooling synonymous sites on estimates of γ is small, as in both cases mkprf uses only the information from fixed and polymorphic sites at candidate *cis*-regulatory regions in order to estimate the strength of selection.
(3.11 MB TIF)

Figure S4 The effect of using a fixed variance of 8 vs. no fixed variance on the prior distribution of γ in candidate *cis*-regulatory regions. The probability of negative selection (top) is reduced above ∼20%, while the probability of positive selection (middle) is reduced above ∼50% when the variance is unfixed. Estimates of mean γ (bottom) demonstrate how not fixing the variance shrinks the estimate of the selection coefficient by restricting the size of the parameter space being explored.
(2.74 MB TIF)

Figure S5 The effects of applying a demographic correction in mkprf on the inference of selection in candidate *cis*-regulatory regions in African Americans. The inclusion of a demographic model increases the variance in the posterior distribution of γ, which widens the distribution of estimated values of γ at a locus, inflating the probability that γ<−0.5 (top left) and the probability that γ>0.5 (bottom left). The probability that γ<0 is unaffected by a demographic correction in candidate *cis*-regulatory regions (bottom right). Estimates of the population scaled selection coefficient (γ = 2N_e_s) are scaled by the current effective population size (N_c_) under a model of population expansion, and by the time-averaged effective population size (N_0_) under a model of constant population size. In African Americans N_c_>N_0_, resulting in a wider distribution of γ (top right) when a demographic correction is applied.
(8.96 MB TIF)

Figure S6 The effects of applying a demographic correction in mkprf on the inference of selection on neutral loci simulated under a model of population expansion inferred from synonymous sites in African Americans. The inclusion of a demographic model increases the variance in the posterior distribution of γ, which widens the distribution of estimated values of γ at a locus, inflating the probability that γ<−0.5 (top left) and the probability that γ>0.5 (bottom left). The overall probability that γ<0 on neutral loci is slightly reduced at higher probabilities after correcting for demography.
(6.22 MB TIF)

Figure S7 Correlations between simulated and estimated mean γ obtained from mkprf (see Table 4 in the main text). Kendall's tau rank correlation tests all have p-values<10^−16^ (except for neut). Loci simulated under positive selection show a stronger correlation than loci simulated under negative selection in the neut+del+pos dataset: *tau* = 0.38 for positively selected loci (*p*<10^−16^), and *tau* = 0.071 for negatively selected loci (*p* = 6.6×10^−3^). However, in the neut+stdel+pos *tau* = 0.27 for positively selected loci (*p*<10^−16^), and *tau* = 0.33 for negatively selected loci (*p*<10^−16^).
(7.47 MB TIF)

Figure S8 The relationship between the total number of sites resequenced in candidate *cis*-regulatory regions (total sites), and the total number of fixed and polymorphic sites (informative sites). We find a significant correlation between estimates of γ and total sites when all genes are considered (Kendall's *tau* = 0.043, *p* = 3.3×10^−11^). The correlation disappears if we condition on genes having at least 4 informative sites (*tau* = −0.0048, *p* = 0.60).
(3.98 MB TIF)

Figure S9 Distribution of mean γ estimated with mkprf for loci simulated under negative (top), positive (middle), and neutral evolution (bottom) in the neut+del+pos dataset (see Table 4 in the main text). “Informative” genes are those with at least 4 fixed or polymorphic sites, and “noninformative” genes are those with <4 fixed or polymorphic sites. Most of the distributions are not significantly different (Mann-Whitney U-tests, *p*>0.05), with the exception of neutral loci where informative genes have a slightly higher mean γ (0.69 vs 0.61, *p* = 0.02). We obtain similar results for the neut+stdel+pos dataset. We note that genes simulated under smaller values of γ are less likely to be in the informative class.
(5.82 MB TIF)

Figure S10 Distribution of the number of sites resequenced in candidate *cis*-regulatory and nonsynonymous sites.
(3.98 MB TIF)

Figure S11 Distribution of estimated mean γ for simulated neutral and positively selected loci analyzed in mkprf with an increasing number of loci simulated under negative selection (see Table 4 in the main text) when there is no fixed variance on the prior distribution of γ. The distributions are significantly different between the neut+pos and the neut+stdel+pos for positively selected loci (Mann-Whitney *U*-test, *p* -values<10^−16^), and are significantly different between all datasets for neutral loci (*p* -values<10^−16^).
(5.97 MB TIF)

Figure S12 The effect of analyzing different classes of sites in a separate, independent run of mkprf vs. a concurrent run including all classes of sites. Simulations suggest that the difference across runs is due to the effects of varying degrees of selection in the background loci, which if not controlled for may be a confounding factor when comparing the extent of natural selection between different runs of mkprf.
(6.87 MB TIF)

Figure S13 Boxplots showing the log of the ratio of the number of polymorphisms to the number of human-chimpanzee fixed differences for unfiltered and HMCS simulated data.
(2.24 MB TIF)

Figure S14 The distribution of L_I_ for the simulated HMCS and unfiltered datasets.
(2.24 MB TIF)

Figure S15 The relationship between GC content and the probability of positive and negative selection in candidate *cis*-regulatory regions, nonsynonymous, and synonymous sites in AAs. GC content in candidate *cis*-regulatory regions shows a weak, but significant negative rank correlation with the probability of negative selection (Kendall's *tau* = −0.014, *p* = 0.029), and a weak, but significant positive rank correlation with the probability of positive selection (*tau* = 0.013, *p* = 0.043). The correlation between GC content and selection on nonsynonymous sites also appears to be weak (negative selection: *tau* = −0.024, *p* = 4.8×10^−4^; positive selection: *tau* = 0.025, *p* = 1.9×10^−4^), similarly for synonymous sites (negative selection: *tau* = −0.021, *p* = 0.0033; positive selection: *tau* = 0.016, *p* = 0.024). Results are similar in EAs.
(9.33 MB TIF)

Figure S16 Flowchart of the bioinformatic pipeline.
(9.44 MB TIF)

Table S1 McDonald-Kreitman tables for candidate *cis*-regulatory regions in AAs.
(0.24 MB TXT)

Table S2 McDonald-Kreitman tables for candidate *cis*-regulatory regions in EAs.
(0.24 MB TXT)

Table S3 McDonald-Kreitman tables for protein-coding regions in AAs.
(0.33 MB TXT)

Table S4 McDonald-Kreitman tables for protein-coding regions in EAs.
(0.33 MB TXT)

Table S5 mkprf results for candidate *cis*-regulatory regions in AAs.
(1.08 MB TXT)

Table S6 mkprf results for candidate *cis*-regulatory regions in EAs.
(1.07 MB TXT)

Table S7 mkprf results for nonsynonymous sites in AAs.
(0.98 MB TXT)

Table S8 mkprf results for nonsynonymous sites in EAs.
(0.97 MB TXT)

Table S9 GeneAtlas2 results for candidate *cis*-regulatory regions.
(0.05 MB XLS)

Table S10 GeneAtlas2 results for protein coding regions.
(0.05 MB XLS)

Table S11 Gene Ontology data for candidate *cis*-regulatory regions.
(0.12 MB XLS)

Table S12 Gene Ontology data for nonsynonymous sites.
(0.12 MB XLS)

Table S13 Summary statistics for the log of the ratio of polymorphism/divergence in simulated human-mouse conserved sequences versus unfiltered sequences.
(0.04 MB PDF)

Table S14 Summary statistics for the distribution of the log of the neutrality index for simulated human-mouse conserved sequences versus unfiltered sequences.
(0.04 MB PDF)

Table S15 Proportion of simulations with no polymorphisms or human-chimpanzee fixed differences in simulated human-mouse conserved sequences and unfiltered sequences.
(0.04 MB PDF)

Text S1 Supplemental methods.
(0.11 MB PDF)

## References

[1] King MC, Wilson AC (1975). Evolution at two levels in humans and chimpanzees.. Science.

[2] Andolfatto P (2005). Adaptive evolution of non-coding DNA in Drosophila.. Nature.

[3] Haddrill PR, Bachtrog D, Andolfatto P (2008). Positive and negative selection on noncoding DNA in *Drosophila simulans*.. Mol Biol Evol.

[4] Gaffney DJ, Keightley PD (2005). The scale of mutational variation in the murid genome.. Genome Res.

[5] Taylor MS, Kai C, Kawai J, Carninci P, Hayashizaki Y (2006). Heterotachy in mammalian promoter evolution.. PLoS Genet.

[6] Bush EC, Lahn BT (2005). Selective constraint on noncoding regions of hominid genomes.. PLoS Comput Biol.

[7] Hughes AL, Packer B, Welch R, Bergen AW, Chanock SJ (2005). Effects of natural selection on interpopulation divergence at polymorphic sites in human protein-coding Loci.. Genetics.

[8] Keightley PD, Kryukov GV, Sunyaev S, Halligan DL, Gaffney DJ (2005). Evolutionary constraints in conserved nongenic sequences of mammals.. Genome Res.

[9] Osada N, Hirata M, Tanuma R, Kusuda J, Hida M (2005). Substitution rate and structural divergence of 5′UTR evolution: comparative analysis between human and cynomolgus monkey cDNAs.. Mol Biol Evol.

[10] Drake JA, Bird C, Nemesh J, Thomas DJ, Newton-Cheh C (2006). Conserved noncoding sequences are selectively constrained and not mutation cold spots.. Nat Genet.

[11] Pollard KS, Salama SR, King B, Kern AD, Dreszer T (2006). Forces shaping the fastest evolving regions in the human genome.. PLoS Genet.

[12] Prabhakar S, Noonan JP, Paabo S, Rubin EM (2006). Accelerated evolution of conserved noncoding sequences in humans.. Science.

[13] Bird CP, Stranger BE, Liu M, Thomas DJ, Ingle CE (2007). Fast-evolving non-coding sequences in the human genome.. Genome Biol.

[14] Asthana S, Noble WS, Kryukov G, Grant CE, Sunyaev S (2007). Widely distributed noncoding purifying selection in the human genome.. Proc Natl Acad Sci U S A.

[15] Kim SY, Pritchard JK (2007). Adaptive Evolution of Conserved Noncoding Elements in Mammals.. PLoS Genet.

[16] Haygood R, Fedrigo O, Hanson B, Yokoyama KD, Wray GA (2007). Promoter regions of many neural- and nutrition-related genes have experienced positive selection during human evolution.. Nat Genet.

[17] Sethupathy P, Giang H, Plotkin JB, Hannenhalli S (2008). Genome-wide analysis of natural selection on human *cis*-elements.. PLoS ONE.

[18] Gaffney DJ, Blekhman R, Majewski J (2008). Selective constraints in experimentally defined primate regulatory regions.. PLoS Genet.

[19] Kudaravalli S, Veyrieras JB, Stranger BE, Dermitzakis ET, Pritchard JK (2009). Gene expression levels are a target of recent natural selection in the human genome.. Mol Biol Evol.

[20] Enard W, Khaitovich P, Klose J, Zollner S, Heissig F (2002). Intra- and interspecific variation in primate gene expression patterns.. Science.

[21] Caceres M, Lachuer J, Zapala MA, Redmond JC, Kudo L (2003). Elevated gene expression levels distinguish human from non-human primate brains.. Proc Natl Acad Sci U S A.

[22] Hsieh WP, Chu TM, Wolfinger RD, Gibson G (2003). Mixed-model reanalysis of primate data suggests tissue and species biases in oligonucleotide-based gene expression profiles.. Genetics.

[23] Khaitovich P, Muetzel B, She X, Lachmann M, Hellmann I (2004). Regional patterns of gene expression in human and chimpanzee brains.. Genome Res.

[24] Khaitovich P, Hellmann I, Enard W, Nowick K, Leinweber M (2005). Parallel patterns of evolution in the genomes and transcriptomes of humans and chimpanzees.. Science.

[25] Blekhman R, Oshlack A, Chabot AE, Smyth GK, Gilad Y (2008). Gene regulation in primates evolves under tissue-specific selection pressures.. PLoS Genet.

[26] Rockman MV, Wray GA (2002). Abundant raw material for *cis*-regulatory evolution in humans.. Mol Biol Evol.

[27] Stranger BE, Forrest MS, Clark AG, Minichiello MJ, Deutsch S (2005). Genome-Wide Associations of Gene Expression Variation in Humans.. PLoS Genet.

[28] Tompa M, Li N, Bailey TL, Church GM, De Moor B (2005). Assessing computational tools for the discovery of transcription factor binding sites.. Nat Biotechnol.

[29] Pennacchio LA, Ahituv N, Moses AM, Prabhakar S, Nobrega MA (2006). In vivo enhancer analysis of human conserved non-coding sequences.. Nature.

[30] Prabhakar S, Poulin F, Shoukry M, Afzal V, Rubin EM (2006). Close sequence comparisons are sufficient to identify human *cis*-regulatory elements.. Genome Res.

[31] The ENCODE Project Consortium (2007). Identification and analysis of functional elements in 1% of the human genome by the ENCODE pilot project.. Nature.

[32] Boyko AR, Williamson SH, Indap AR, Degenhardt JD, Hernandez RD (2008). Assessing the evolutionary impact of amino acid mutations in the human genome.. PLoS Genet.

[33] Lohmueller KE, Indap A, Schmidt S, Boyko AR, Hernandez RH (2008). Proportionally more deleterious genetic variation in European than in African populations.. Nature.

[34] Veyrieras JB, Kudaravalli S, Kim SY, Dermitzakis ET, Gilad Y (2008). High-resolution mapping of expression-QTLs yields insight into human gene regulation.. PLoS Genet.

[35] Bustamante CD, Fledel-Alon A, Williamson S, Nielsen R, Hubisz MT (2005). Natural selection on protein-coding genes in the human genome.. Nature.

[36] Bustamante CD, Nielsen R, Sawyer SA, Olsen KM, Purugganan MD (2002). The cost of inbreeding in Arabidopsis.. Nature.

[37] Hernandez RD (2008). A flexible forward simulator for populations subject to selection and demography.. Bioinformatics.

[38] Su AI, Wiltshire T, Batalov S, Lapp H, Ching KA (2004). A gene atlas of the mouse and human protein-encoding transcriptomes.. Proc Natl Acad Sci U S A.

[39] Yang J, Su AI, Li WH (2005). Gene expression evolves faster in narrowly than in broadly expressed mammalian genes.. Mol Biol Evol.

[40] Liao BY, Zhang J (2006). Low rates of expression profile divergence in highly expressed genes and tissue-specific genes during Mammalian evolution.. Mol Biol Evol.

[41] Yanai I, Benjamin H, Shmoish M, Chalifa-Caspi V, Shklar M (2005). Genome-wide midrange transcription profiles reveal expression level relationships in human tissue specification.. Bioinformatics.

[42] Finlay BL, Darlington RB (1995). Linked regularities in the development and evolution of mammalian brains.. Science.

[43] Sherwood CC (2005). Comparative anatomy of the facial motor nucleus in mammals, with an analysis of neuron numbers in primates.. Anat Rec A Discov Mol Cell Evol Biol.

[44] Strand AD, Aragaki AK, Baquet ZC, Hodges A, Cunningham P (2007). Conservation of regional gene expression in mouse and human brain.. PLoS Genet.

[45] Nielsen R, Bustamante C, Clark AG, Glanowski S, Sackton TB (2005). A scan for positively selected genes in the genomes of humans and chimpanzees.. PLoS Biol.

[46] Gibbs RA, Rogers J, Katze MG, Bumgarner R, Weinstock GM (2007). Evolutionary and biomedical insights from the rhesus macaque genome.. Science.

[47] Nielsen R, Hellmann I, Hubisz M, Bustamante C, Clark AG (2007). Recent and ongoing selection in the human genome.. Nat Rev Genet.

[48] Ashburner M, Ball CA, Blake JA, Botstein D, Butler H (2000). Gene ontology: tool for the unification of biology. The Gene Ontology Consortium.. Nat Genet.

[49] Voight BF, Kudaravalli S, Wen X, Pritchard JK (2006). A map of recent positive selection in the human genome.. PLoS Biol.

[50] Wang ET, Kodama G, Baldi P, Moyzis RK (2006). Global landscape of recent inferred Darwinian selection for *Homo sapiens*.. Proc Natl Acad Sci U S A.

[51] Smyth I, Du X, Taylor MS, Justice MJ, Beutler B (2004). The extracellular matrix gene Frem1 is essential for the normal adhesion of the embryonic epidermis.. Proc Natl Acad Sci U S A.

[52] Blekhman R, Man O, Herrmann L, Boyko AR, Indap A (2008). Natural selection on genes that underlie human disease susceptibility.. Curr Biol.

[53] Becker KG, Barnes KC, Bright TJ, Wang SA (2004). The genetic association database.. Nat Genet.

[54] Knight JC (2005). Regulatory polymorphisms underlying complex disease traits.. J Mol Med.

[55] Chamary JV, Parmley JL, Hurst LD (2006). Hearing silence: non-neutral evolution at synonymous sites in mammals.. Nat Rev Genet.

[56] Kent WJ, Sugnet CW, Furey TS, Roskin KM, Pringle TH (2002). The human genome browser at UCSC.. Genome Res.

[57] Altschul SF, Gish W, Miller W, Myers EW, Lipman DJ (1990). Basic local alignment search tool.. J Mol Biol.

[58] Kent WJ (2002). BLAT–the BLAST-like alignment tool.. Genome Res.

[59] Karolchik D, Baertsch R, Diekhans M, Furey TS, Hinrichs A (2003). The UCSC Genome Browser Database.. Nucleic Acids Res.

[60] Barrier M, Bustamante CD, Yu J, Purugganan MD (2003). Selection on rapidly evolving proteins in the Arabidopsis genome.. Genetics.

[61] Williamson SH, Hernandez R, Fledel-Alon A, Zhu L, Nielsen R (2005). Simultaneous inference of selection and population growth from patterns of variation in the human genome.. Proc Natl Acad Sci U S A.

[62] Li YF, Costello JC, Holloway AK, Hahn MW (2008). “Reverse ecology” and the power of population genomics.. Evolution.

[63] Benjamini Y, Hochberg Y (1995). Controlling the false discovery rate: a practical and powerful approach to multiple testing.. Journal of the Royal Statistical Society Series B.

[64] Hernandez RD, Williamson SH, Bustamante CD (2007). Context dependence, ancestral misidentification, and spurious signatures of natural selection.. Mol Biol Evol.

