# Supplementary figures and images for: Evolutionary Processes Acting on Candidate cis-Regulatory Regions in Humans Inferred from Patterns of Polymorphism and Divergence
Source: PLoS Genet. 2009 Aug 7;5(8):e1000592. doi: 10.1371/journal.pgen.1000592 (PMC2714078; doi:10.1371/journal.pgen.1000592)

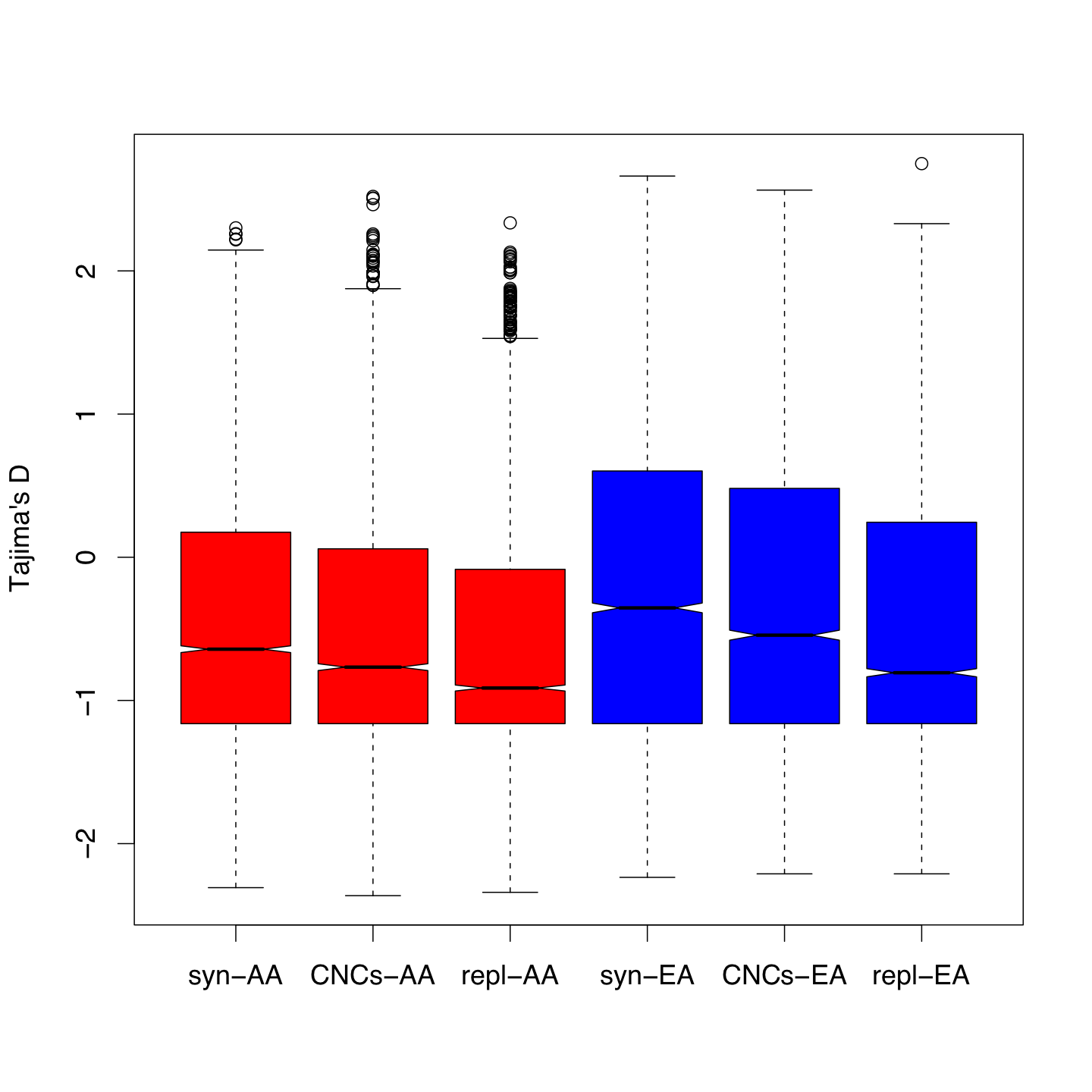

Supplement: Figure S1 — Estimates of Tajima's D for different categories of sites; syn = synonymous sites, CNCs = conserved non-coding sites, repl = nonsynonymous sites. Notches represent the 95% confidence interval for the difference in two medians. (6.22 MB TIF) [file pgen.1000592.s001.tif]

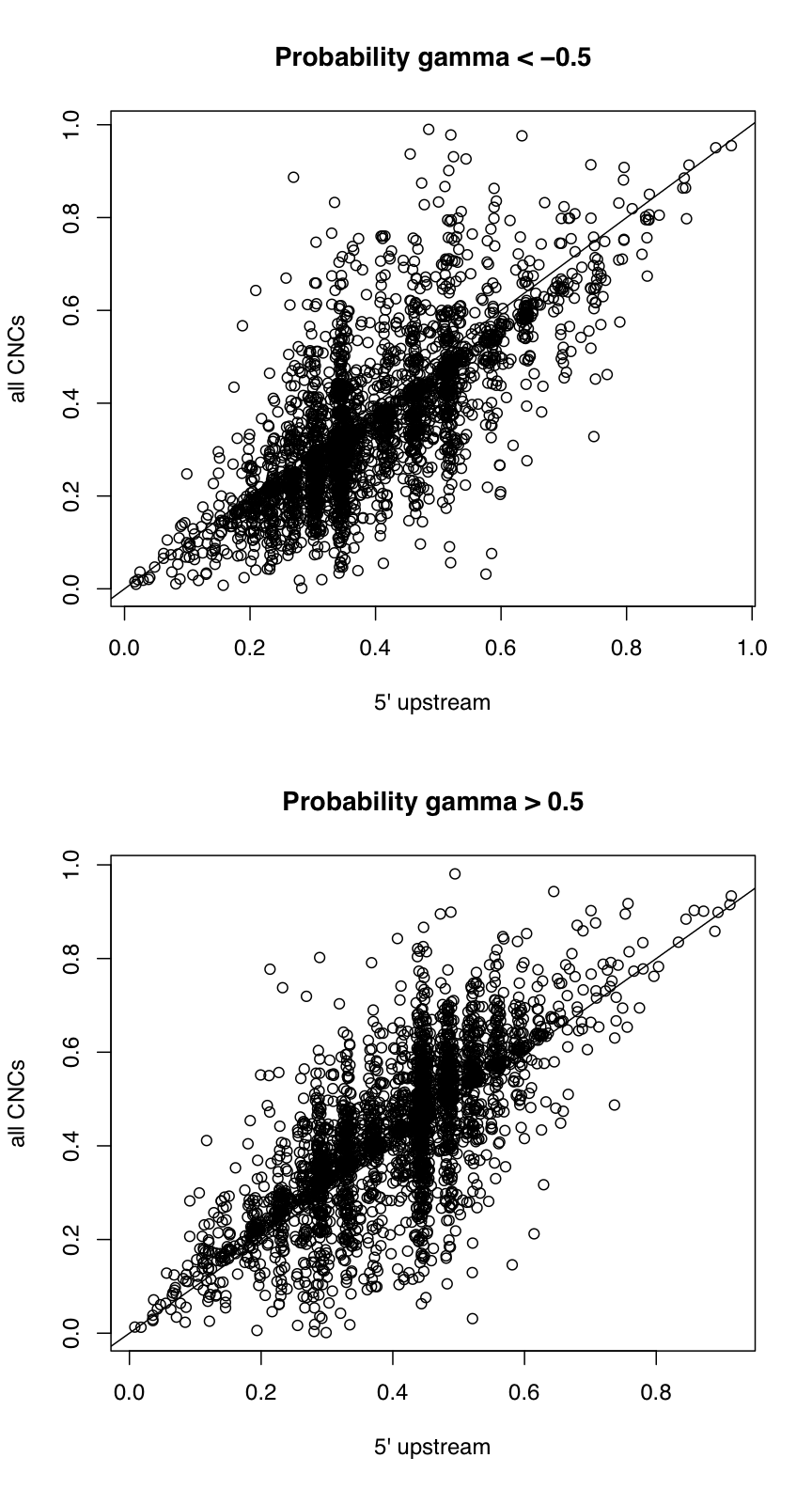

Supplement: Figure S2 — Comparison of the probability of negative selection (above) and the probability of positive selection (below) when only CNCs in the 5′ upstream regions of genes are considered, as compared to when all CNCs are pooled for African Americans in mkprf. (4.11 MB TIF) [file pgen.1000592.s002.tif]

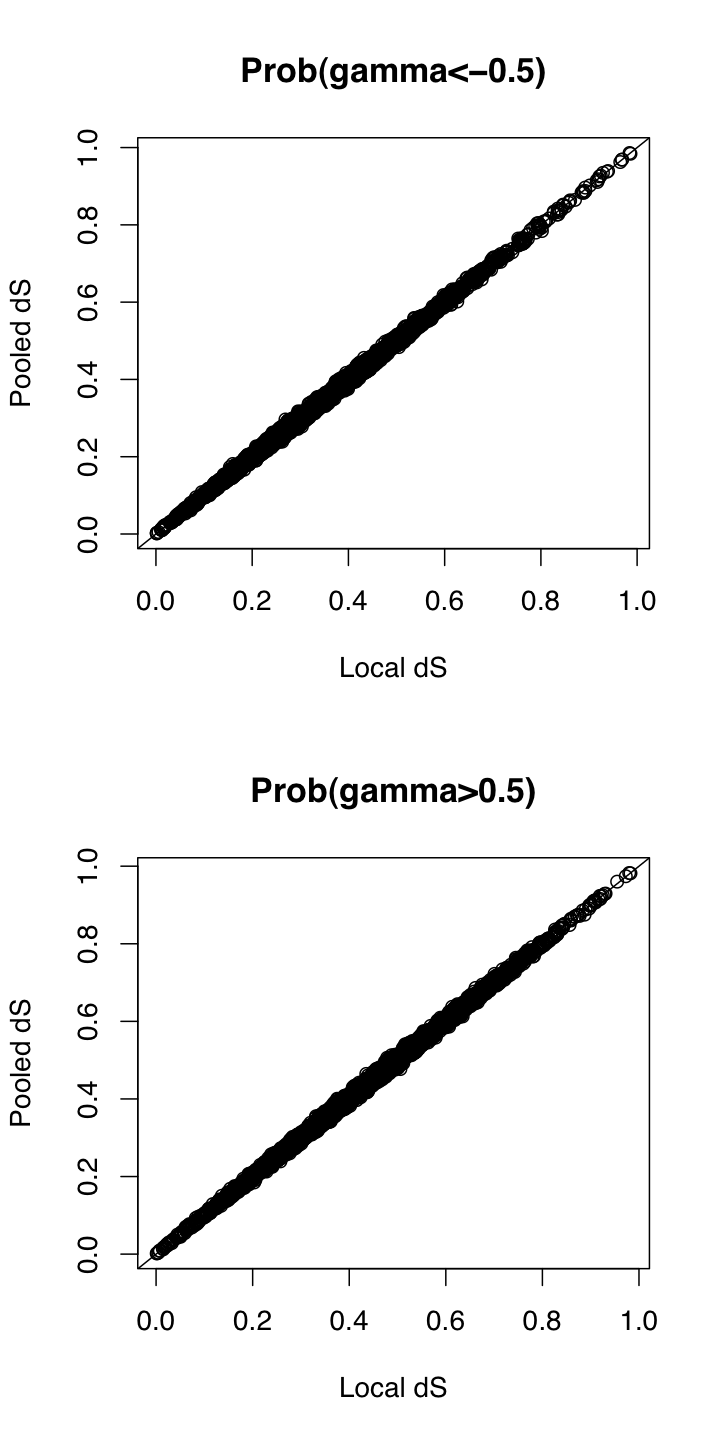

Supplement: Figure S3 — The effect of using pooled vs local synonymous sites on estimates of the probability of negative selection (above) and the probability of positive selection (below) for candidate cis-regulatory regions in the African American sample. The effect of pooling synonymous sites on estimates of γ is small, as in both cases mkprf uses only the information from fixed and polymorphic sites at candidate cis-regulatory regions in order to estimate the strength of selection. (3.11 MB TIF) [file pgen.1000592.s003.tif]

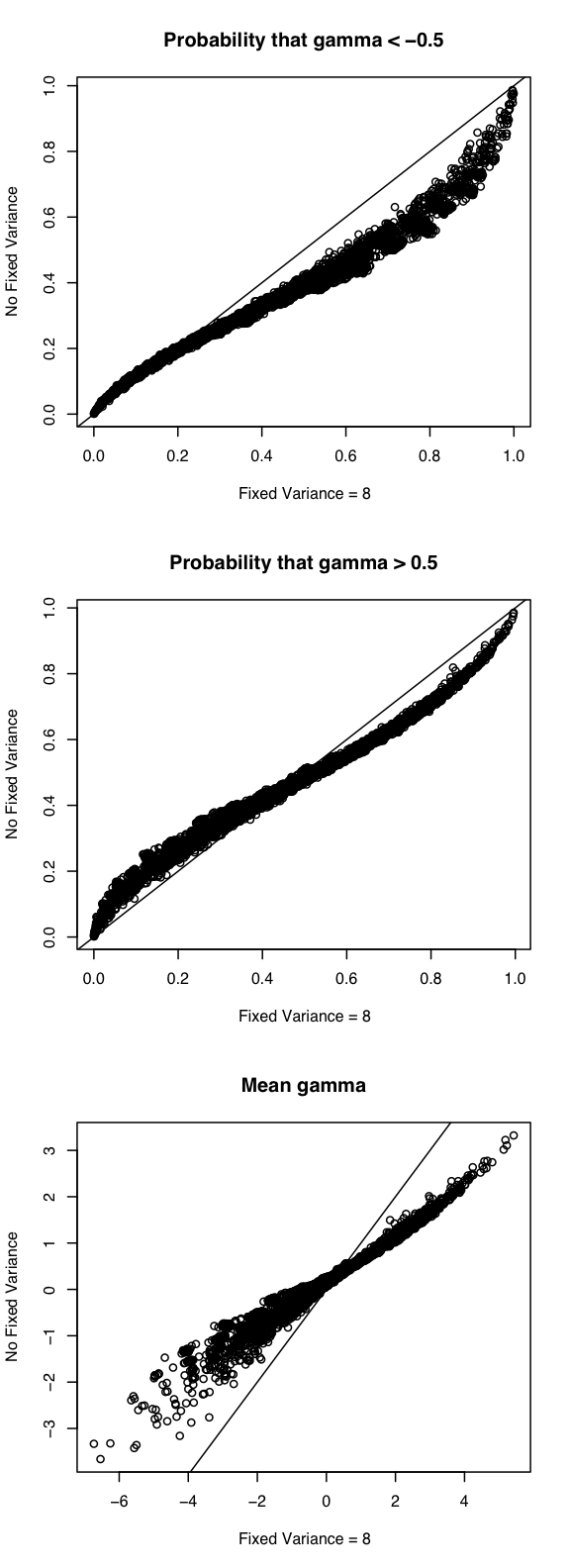

Supplement: Figure S4 — The effect of using a fixed variance of 8 vs. no fixed variance on the prior distribution of γ in candidate cis-regulatory regions. The probability of negative selection (top) is reduced above ∼20%, while the probability of positive selection (middle) is reduced above ∼50% when the variance is unfixed. Estimates of mean γ (bottom) demonstrate how not fixing the variance shrinks the estimate of the selection coefficient by restricting the size of the parameter space being explored. (2.74 MB TIF) [file pgen.1000592.s004.tif]

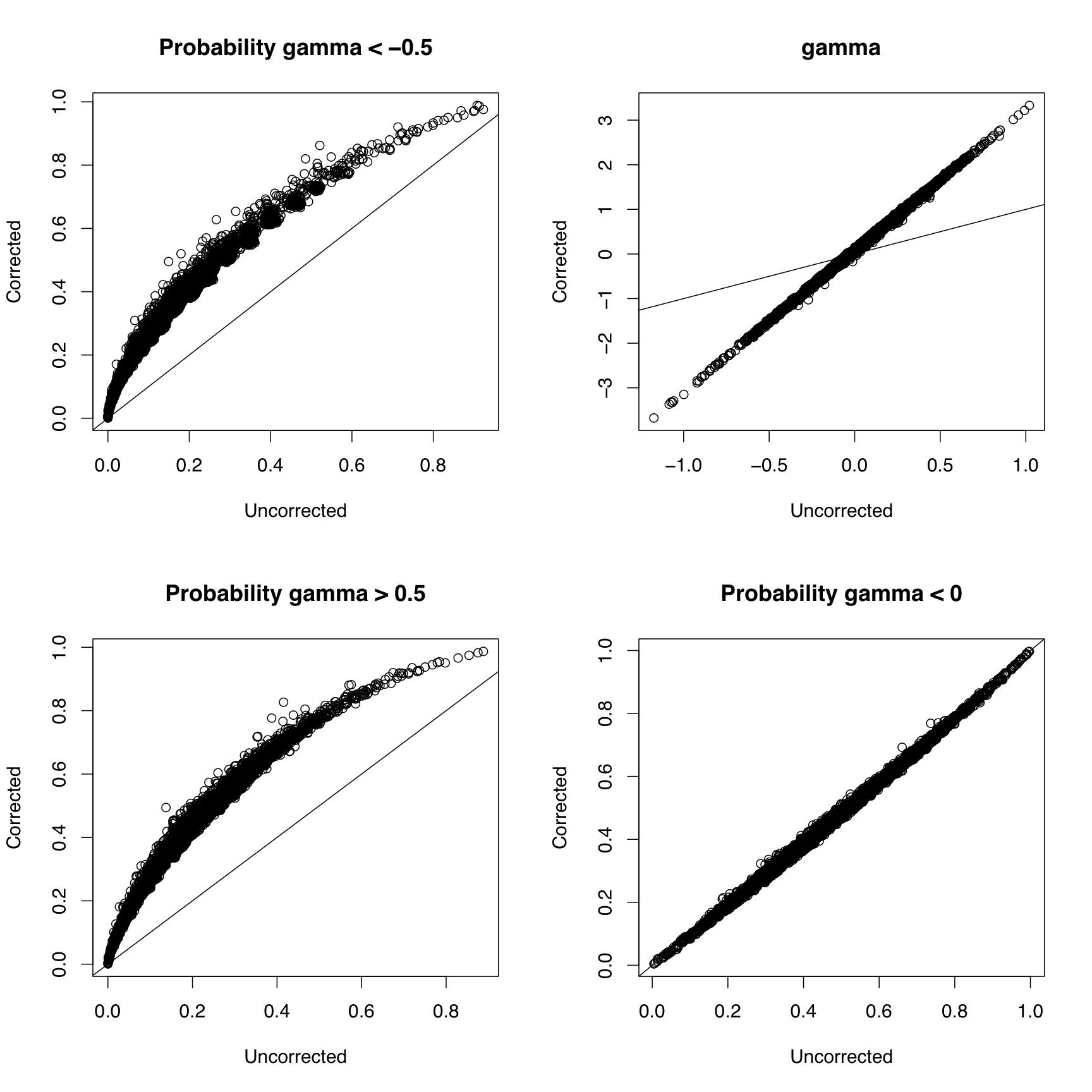

Supplement: Figure S5 — The effects of applying a demographic correction in mkprf on the inference of selection in candidate cis-regulatory regions in African Americans. The inclusion of a demographic model increases the variance in the posterior distribution of γ, which widens the distribution of estimated values of γ at a locus, inflating the probability that γ<−0.5 (top left) and the probability that γ>0.5 (bottom left). The probability that γ<0 is unaffected by a demographic correction in candidate cis-regulatory regions (bottom right). Estimates of the population scaled selection coefficient (γ = 2Nes) are scaled by the current effective population size (Nc) under a model of population expansion, and by the time-averaged effective population size (N0) under a model of constant population size. In African Americans Nc>N0, resulting in a wider distribution of γ (top right) when a demographic correction is applied. (8.96 MB TIF) [file pgen.1000592.s005.tif]

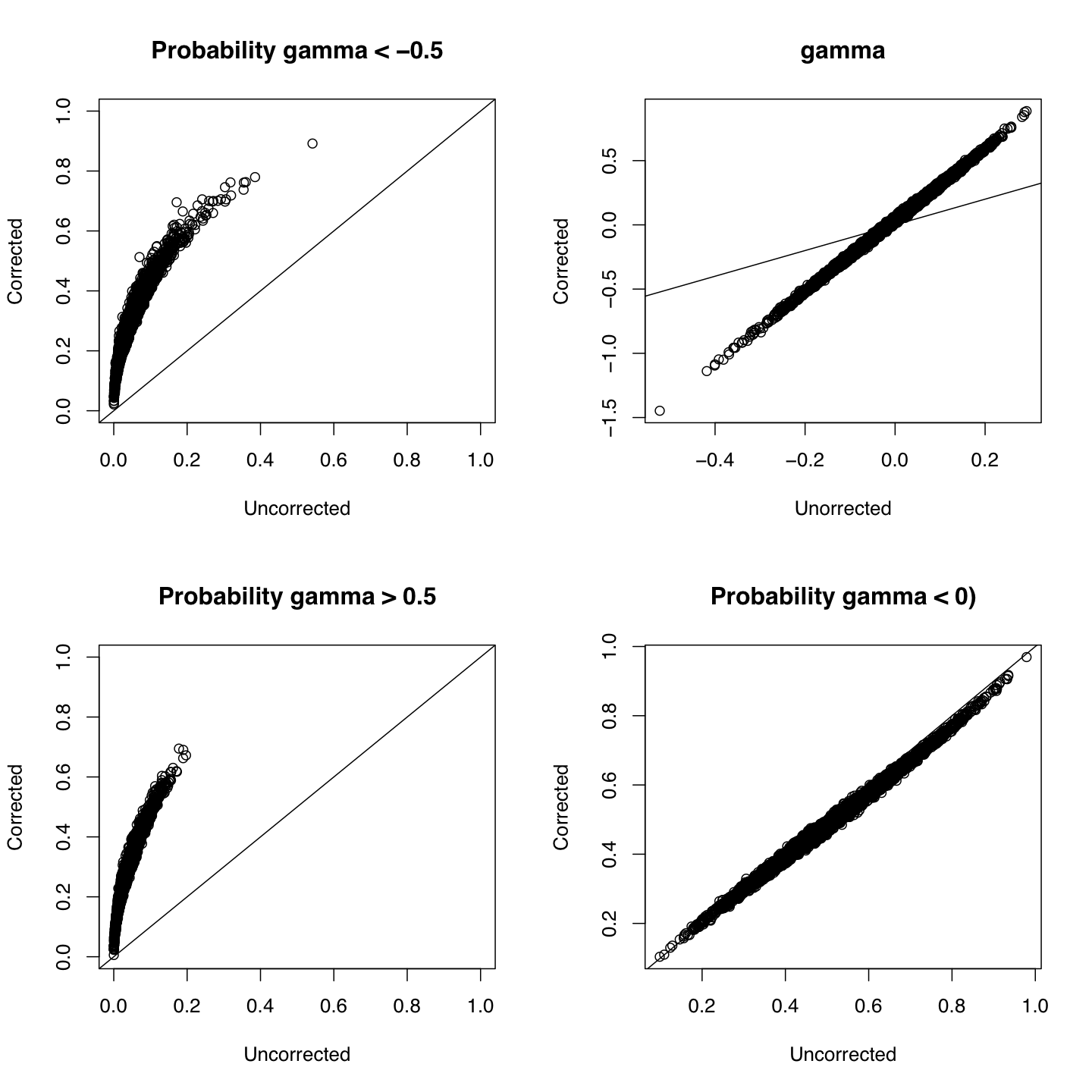

Supplement: Figure S6 — The effects of applying a demographic correction in mkprf on the inference of selection on neutral loci simulated under a model of population expansion inferred from synonymous sites in African Americans. The inclusion of a demographic model increases the variance in the posterior distribution of γ, which widens the distribution of estimated values of γ at a locus, inflating the probability that γ<−0.5 (top left) and the probability that γ>0.5 (bottom left). The overall probability that γ<0 on neutral loci is slightly reduced at higher probabilities after correcting for demography. (6.22 MB TIF) [file pgen.1000592.s006.tif]

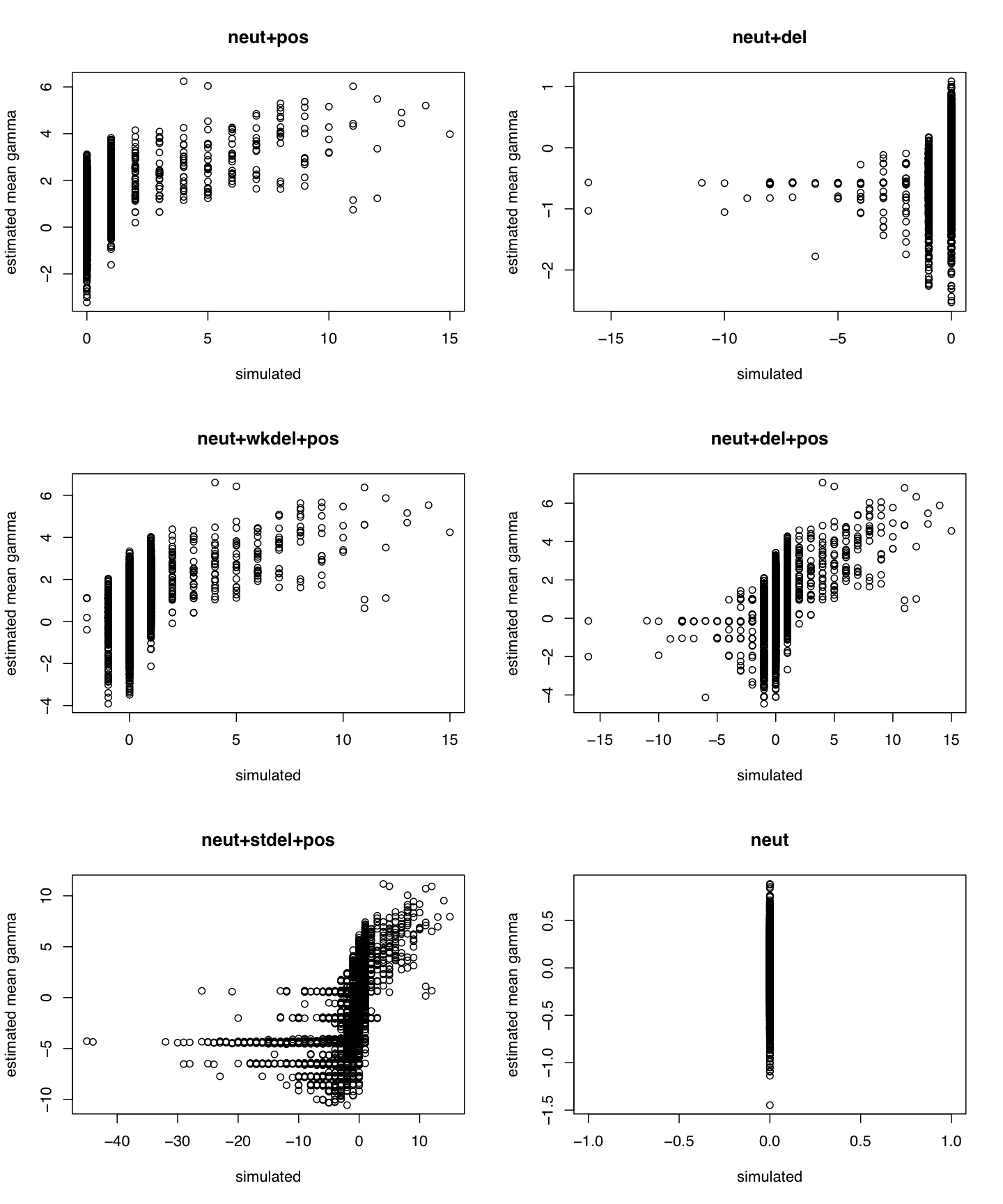

Supplement: Figure S7 — Correlations between simulated and estimated mean γ obtained from mkprf (see Table 4 in the main text). Kendall's tau rank correlation tests all have p-values<10−16 (except for neut). Loci simulated under positive selection show a stronger correlation than loci simulated under negative selection in the neut+del+pos dataset: tau = 0.38 for positively selected loci (p<10−16), and tau = 0.071 for negatively selected loci (p = 6.6×10^−3^). However, in the neut+stdel+pos tau = 0.27 for positively selected loci (p<10−16), and tau = 0.33 for negatively selected loci (p<10−16). (7.47 MB TIF) [file pgen.1000592.s007.tif]

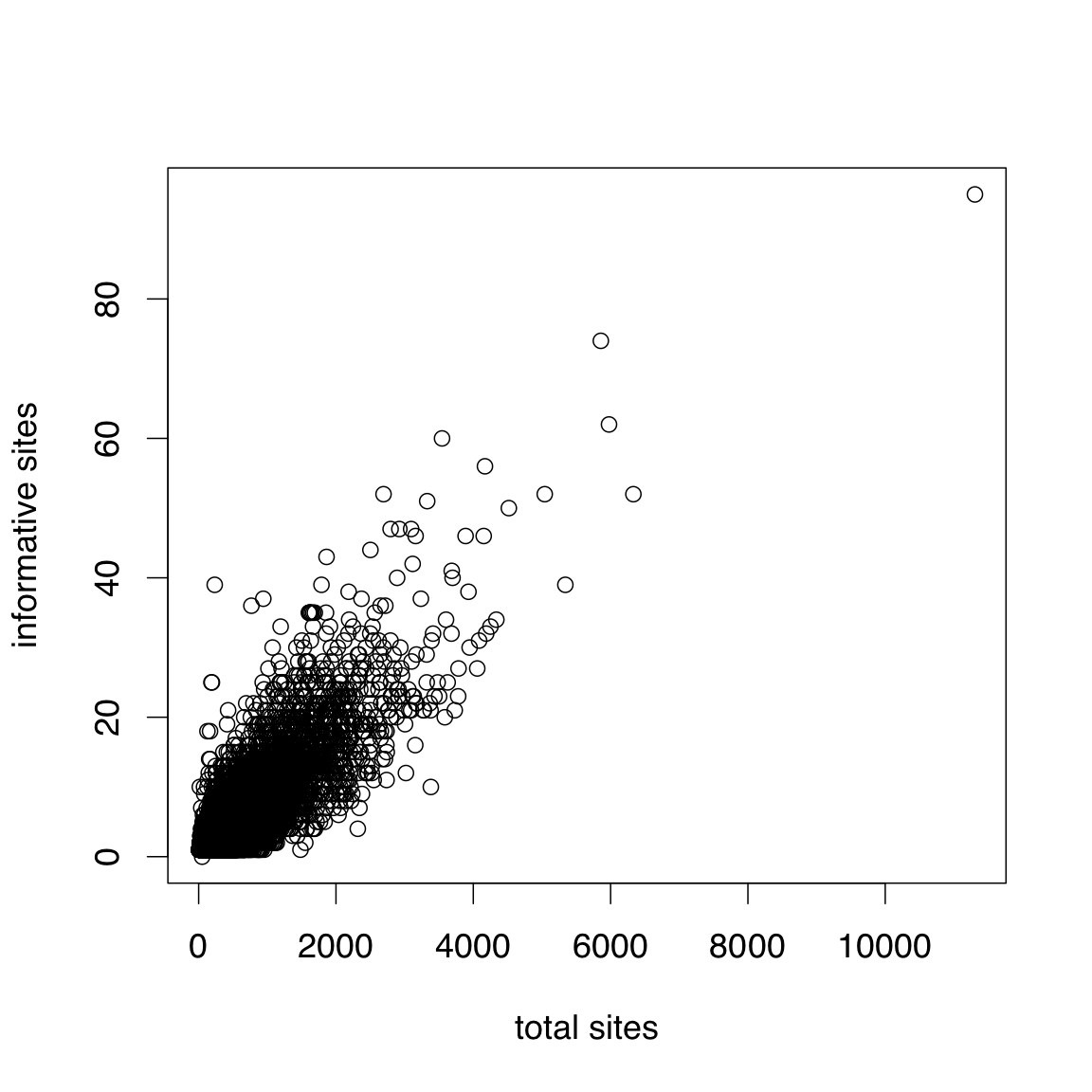

Supplement: Figure S8 — The relationship between the total number of sites resequenced in candidate cis-regulatory regions (total sites), and the total number of fixed and polymorphic sites (informative sites). We find a significant correlation between estimates of γ and total sites when all genes are considered (Kendall's tau = 0.043, p = 3.3×10^−11^). The correlation disappears if we condition on genes having at least 4 informative sites (tau = −0.0048, p = 0.60). (3.98 MB TIF) [file pgen.1000592.s008.tif]

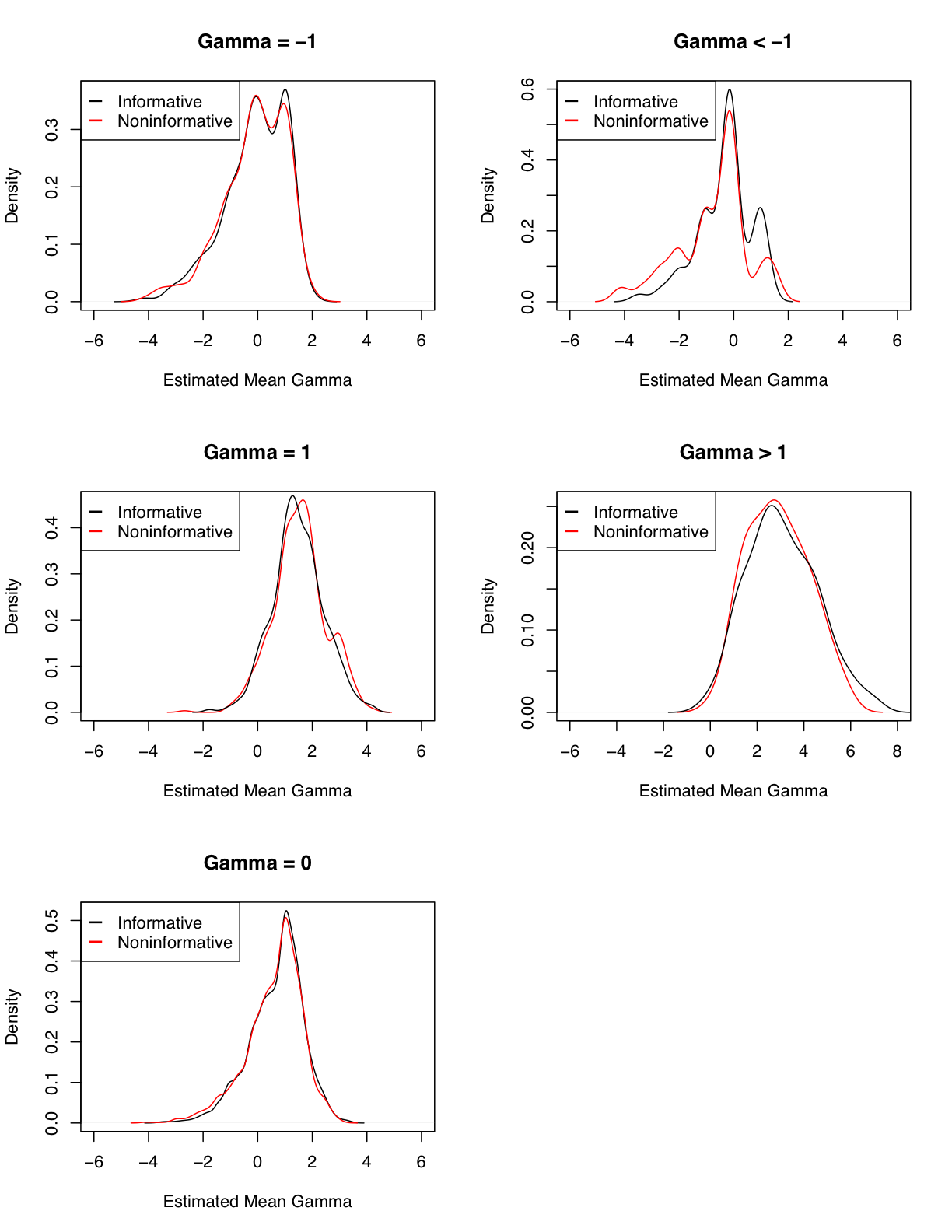

Supplement: Figure S9 — Distribution of mean γ estimated with mkprf for loci simulated under negative (top), positive (middle), and neutral evolution (bottom) in the neut+del+pos dataset (see Table 4 in the main text). “Informative” genes are those with at least 4 fixed or polymorphic sites, and “noninformative” genes are those with <4 fixed or polymorphic sites. Most of the distributions are not significantly different (Mann-Whitney U-tests, p>0.05), with the exception of neutral loci where informative genes have a slightly higher mean γ (0.69 vs 0.61, p = 0.02). We obtain similar results for the neut+stdel+pos dataset. We note that genes simulated under smaller values of γ are less likely to be in the informative class. (5.82 MB TIF) [file pgen.1000592.s009.tif]

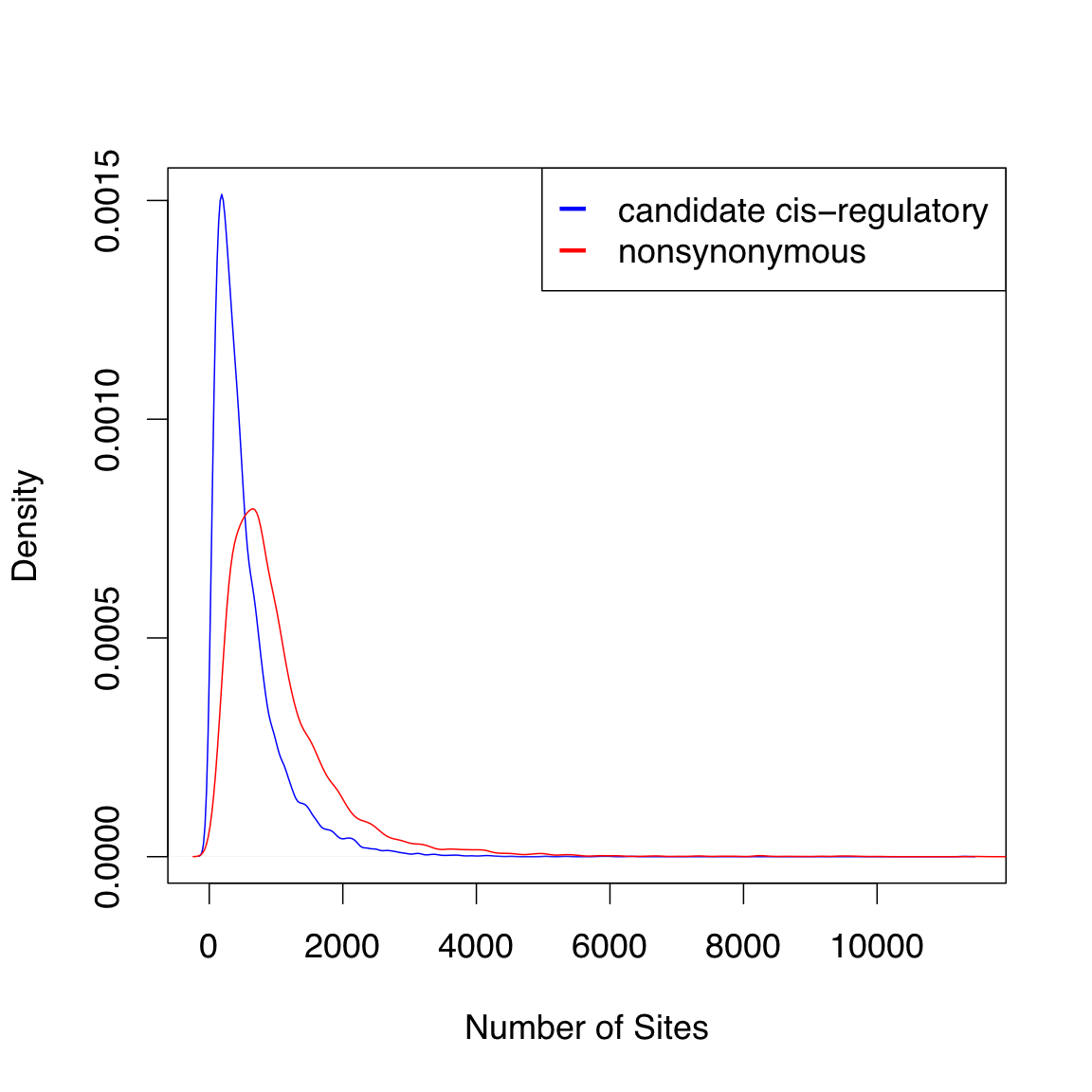

Supplement: Figure S10 — Distribution of the number of sites resequenced in candidate cis-regulatory and nonsynonymous sites. (3.98 MB TIF) [file pgen.1000592.s010.tif]

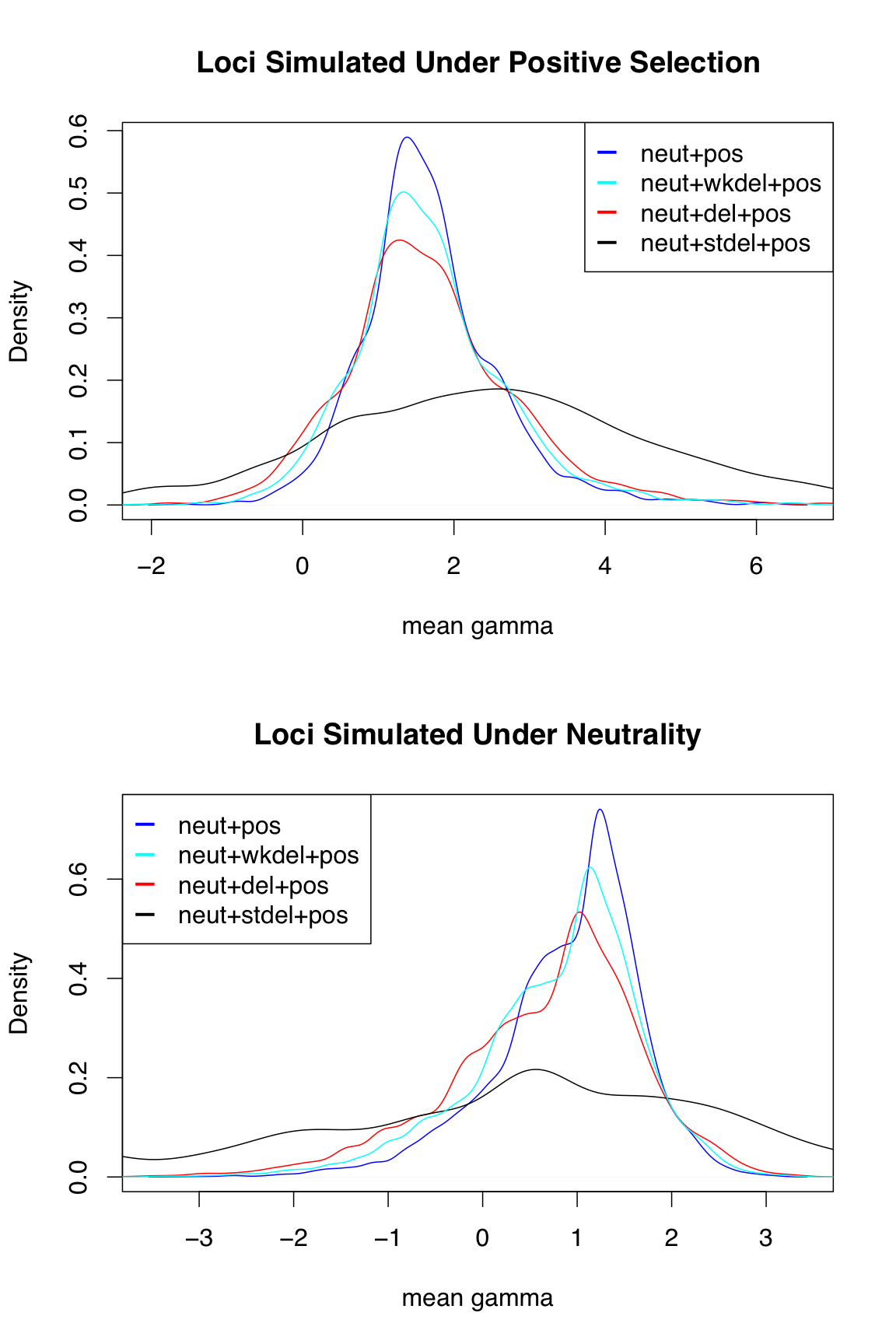

Supplement: Figure S11 — Distribution of estimated mean γ for simulated neutral and positively selected loci analyzed in mkprf with an increasing number of loci simulated under negative selection (see Table 4 in the main text) when there is no fixed variance on the prior distribution of γ. The distributions are significantly different between the neut+pos and the neut+stdel+pos for positively selected loci (Mann-Whitney U-test, p -values<10−16), and are significantly different between all datasets for neutral loci (p -values<10−16). (5.97 MB TIF) [file pgen.1000592.s011.tif]

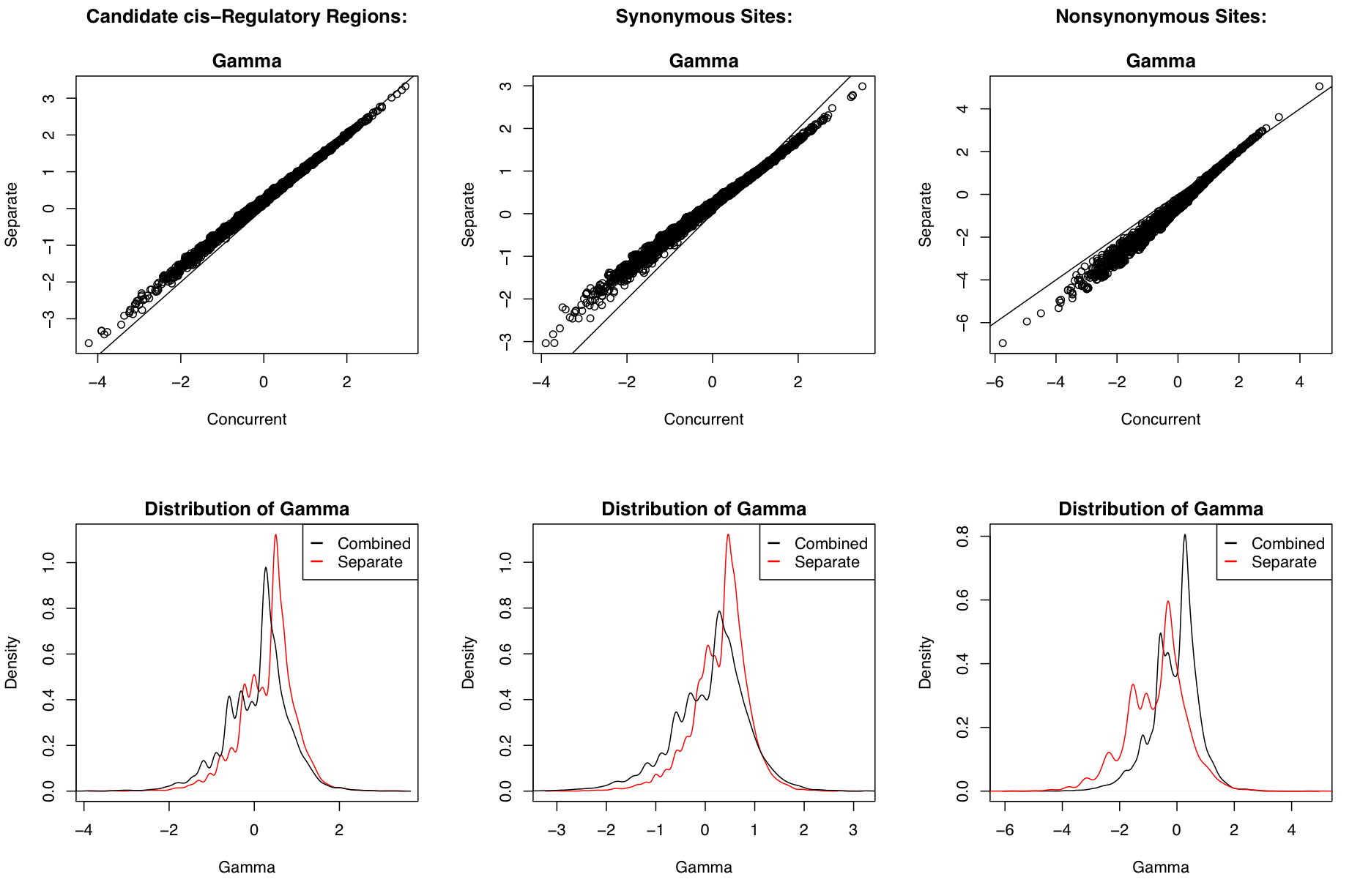

Supplement: Figure S12 — The effect of analyzing different classes of sites in a separate, independent run of mkprf vs. a concurrent run including all classes of sites. Simulations suggest that the difference across runs is due to the effects of varying degrees of selection in the background loci, which if not controlled for may be a confounding factor when comparing the extent of natural selection between different runs of mkprf. (6.87 MB TIF) [file pgen.1000592.s012.tif]

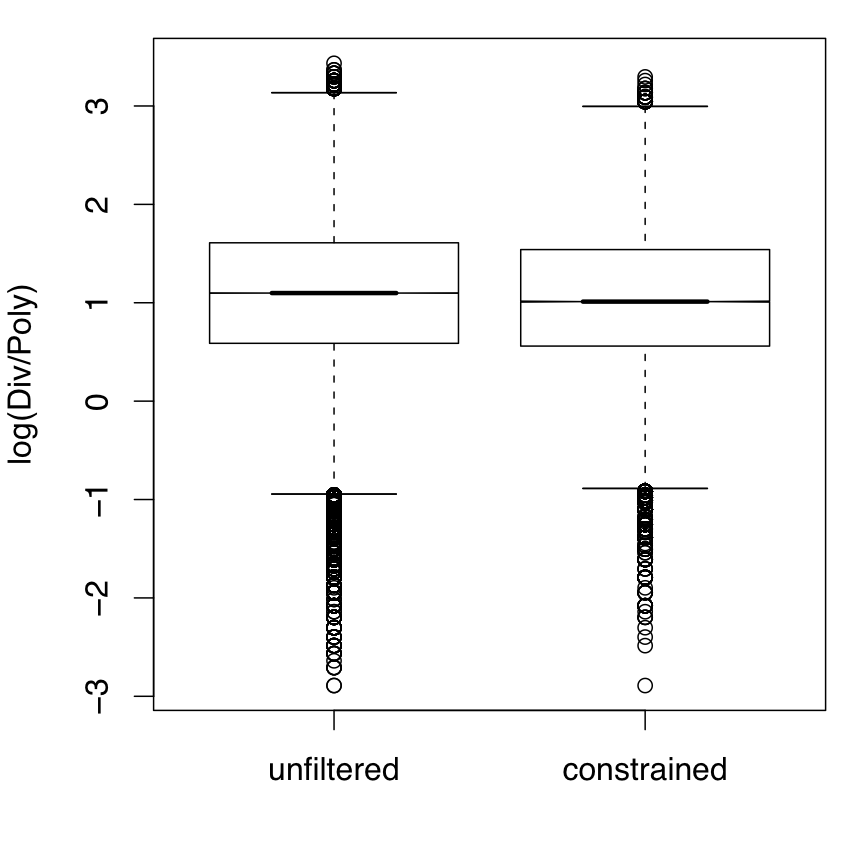

Supplement: Figure S13 — Boxplots showing the log of the ratio of the number of polymorphisms to the number of human-chimpanzee fixed differences for unfiltered and HMCS simulated data. (2.24 MB TIF) [file pgen.1000592.s013.tif]

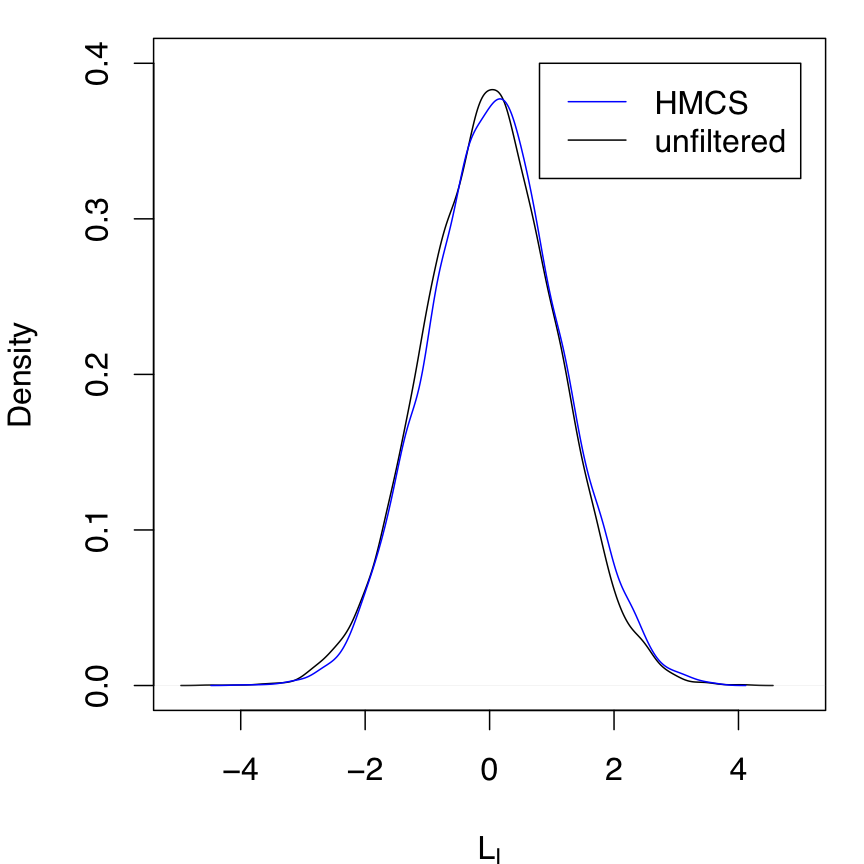

Supplement: Figure S14 — The distribution of LI for the simulated HMCS and unfiltered datasets. (2.24 MB TIF) [file pgen.1000592.s014.tif]

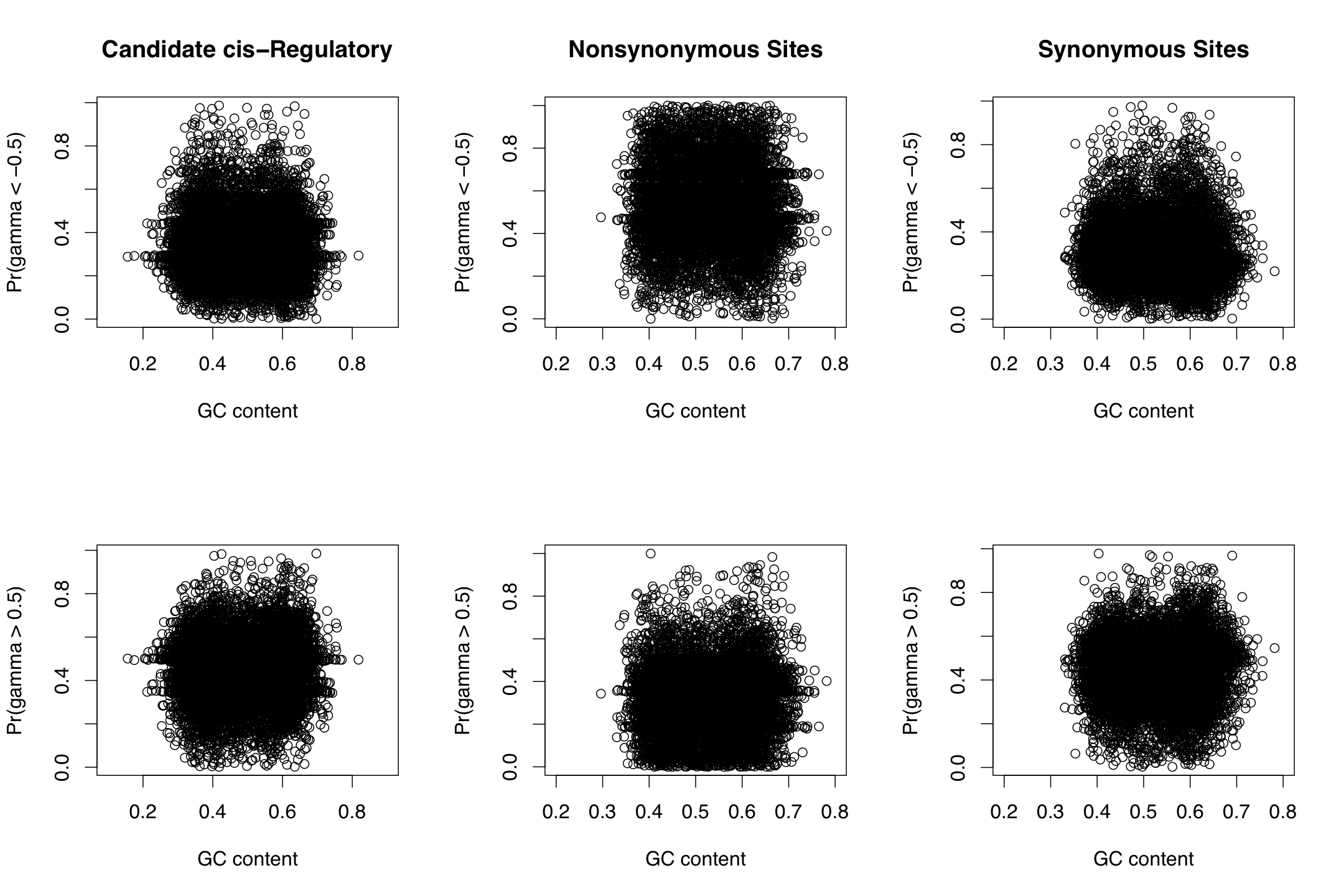

Supplement: Figure S15 — The relationship between GC content and the probability of positive and negative selection in candidate cis-regulatory regions, nonsynonymous, and synonymous sites in AAs. GC content in candidate cis-regulatory regions shows a weak, but significant negative rank correlation with the probability of negative selection (Kendall's tau = −0.014, p = 0.029), and a weak, but significant positive rank correlation with the probability of positive selection (tau = 0.013, p = 0.043). The correlation between GC content and selection on nonsynonymous sites also appears to be weak (negative selection: tau = −0.024, p = 4.8×10^−4^; positive selection: tau = 0.025, p = 1.9×10^−4^), similarly for synonymous sites (negative selection: tau = −0.021, p = 0.0033; positive selection: tau = 0.016, p = 0.024). Results are similar in EAs. (9.33 MB TIF) [file pgen.1000592.s015.tif]

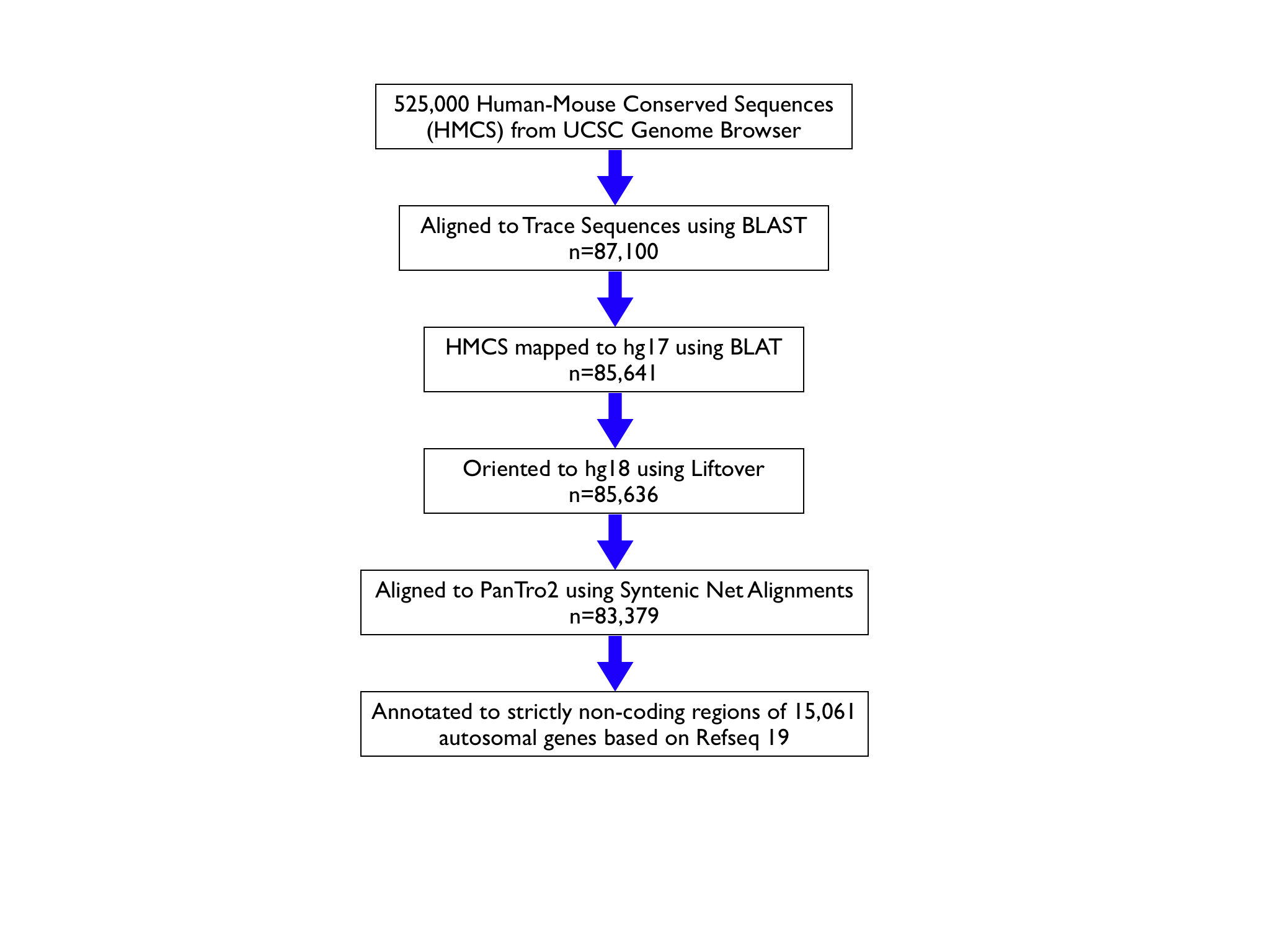

Supplement: Figure S16 — Flowchart of the bioinformatic pipeline. (9.44 MB TIF) [file pgen.1000592.s016.tif]
