# Supplementary material for: Evolutionary Processes Acting on Candidate cis-Regulatory Regions in Humans Inferred from Patterns of Polymorphism and Divergence
Source: PLoS Genet. 2009 Aug 7;5(8):e1000592. doi: 10.1371/journal.pgen.1000592 (PMC2714078; doi:10.1371/journal.pgen.1000592)
Supplement: Table S13 — Summary statistics for the log of the ratio of polymorphism/divergence in simulated human-mouse conserved sequences versus unfiltered sequences. (0.04 MB PDF) [file pgen.1000592.s029.pdf]

Table S13. Summary statistics for the log of polymorphism/divergence ratio in human-mouse conserved sequences (HMCS) versus unfiltered data.

Data	Min	1st Quartile	Median	Mean	3rd Quartile	Max.
HMCS	-3.1350	-1.5040	-0.9808	-1.0220	-0.5108	2.4850
Unfiltered	-3.4340	-1.6090	-1.0990	-1.1040	-0.5878	2.8900
