# Supplementary material for: Evolutionary Processes Acting on Candidate cis-Regulatory Regions in Humans Inferred from Patterns of Polymorphism and Divergence
Source: PLoS Genet. 2009 Aug 7;5(8):e1000592. doi: 10.1371/journal.pgen.1000592 (PMC2714078; doi:10.1371/journal.pgen.1000592)
Supplement: Table S14 — Summary statistics for the distribution of the log of the neutrality index for simulated human-mouse conserved sequences versus unfiltered sequences. (0.04 MB PDF) [file pgen.1000592.s030.pdf]

Table S14. Summary statistics for the distribution of the log of the neutrality index for HMCS versus unfiltered simulated data across 10,000 nonparametric bootstrap draws.

Data	Min.	1st Quartile	Median	Mean	3rd Quartile	Max.
HMCS	-4.02500	-0.64250	0.08701	0.08074	0.78850	3.66400
Unfiltered	-4.51100	-0.69310	0.00000	0.01148	0.71560	4.10500
