# Supplementary material for: Evolutionary Processes Acting on Candidate cis-Regulatory Regions in Humans Inferred from Patterns of Polymorphism and Divergence
Source: PLoS Genet. 2009 Aug 7;5(8):e1000592. doi: 10.1371/journal.pgen.1000592 (PMC2714078; doi:10.1371/journal.pgen.1000592)
Supplement: Table S15 — Proportion of simulations with no polymorphisms or human-chimpanzee fixed differences in simulated human-mouse conserved sequences and unfiltered sequences. (0.04 MB PDF) [file pgen.1000592.s031.pdf]

Table S15. Proportion of simulations with zero polymorphisms (*p*=0) or zero human-chimpanzee fixed differences (*d*=0) in the HMCS and unfiltered data.

Data	*p*=0	*d*=0
HMCS	0.0498	0.0
Unfiltered	0.04996	0.000087
