# Supplementary material for: Evolutionary Processes Acting on Candidate cis-Regulatory Regions in Humans Inferred from Patterns of Polymorphism and Divergence
Source: PLoS Genet. 2009 Aug 7;5(8):e1000592. doi: 10.1371/journal.pgen.1000592 (PMC2714078; doi:10.1371/journal.pgen.1000592)
Supplement: Text S1 — Supplemental methods. (0.11 MB PDF) [file pgen.1000592.s032.pdf]

Text S1 – Coalescent Simulations

We performed extensive coalescent simulations with the program ms [1] to show that the method we employed to infer both positive and negative selection based on levels of polymorphism and divergence with chimpanzee were not biased by the focus on regions of the human genome showing phylogenetic conservation between human and mouse. For these simulations, we generated a sample of 22 orthologous chromosomes (20 representing the human sample, and one representing the chimpanzee and mouse reference genomes) under the standard neutral model of evolution. We assumed that the human and chimpanzee lineages diverged t=5×4N_e generations ago, while primate lineages diverged from rodents t=250×4N_e generations ago. Under standard neutral model assumptions (*i.e.*, in the absence of natural selection), the expected level of nucleotide divergence between human and chimpanzee is 1%, while the divergence between human and mouse is 50% (approximately the values inferred by Hwang and Green [2] from 5.2 Mb of non-coding DNA). A total of 10^6 genes were simulated independently. Those genes with human-mouse divergence in the 1% quantile (*i.e.*, the most conserved genes) were then considered as our human-mouse conserved sequence (HMCS) dataset. The log of the ratio of human polymorphism to human-chimpanzee divergence for the simulated HMCS dataset (R_{con}) was then compared to the corresponding ratio for the non-HMCS dataset (hereafter referred to as the unfiltered data set, R_{unf}) after excluding datasets with either zero polymorphisms or zero fixed differences (the consequences of which are discussed below). As shown in Table S13 and Figure S14, the range of R_{con} is narrower than R_{unf}, with the mean and median of R_{con} slightly shifted toward larger values (*i.e.*, an excess of human polymorphism relative to human-chimpanzee divergence).

Moreover, as our analysis of selection on HMCS is based on the McDonald-Kreitman test, it is necessary to understand the performance of the neutrality index, defined as

$$I = \frac{p_n / d_n}{p_s / d_s}$$

the odds ratio of the McDonald-Kreitman table, where p_n and d_n are the number of polymorphisms and fixed differences (respectively) in the test set (*i.e.*, HMCS), and p_s and d_s are the number of polymorphisms and fixed differences in the reference set (*i.e.*, unfiltered dataset). To normalize this statistic, we consider $L_I = log(I)$. Under the standard neutral model, the distribution of L_I is centered on zero. Values of $L_I > 0$ indicate that there is an excess of polymorphism in the test set, suggesting evidence of negative selection, while values of $L_I < 0$ indicate an a deficiency of polymorphism (corresponding to an excess of fixed differences) in the test set, suggesting the presence of positive selection. We generated an empirical distribution of L_I using 10,000 nonparametric bootstrap draws from both the simulated HMCS dataset and the unfiltered dataset (*i.e.*, 10,000 values of p_n, d_n, p_s, and d_s). For reference, we also generated a corresponding distribution from the unfiltered data (drawing two sets of 10,000 simulations independently). As shown in Figure S15 and the summary statistics in Table S14, L_I is nearly unbiased by conservation at the level of human and mouse, though the mean of L_I for the HMCS dataset is slightly shifted toward larger values (indicating an excess of polymorphism). This suggests that McDonald-Kreitman style tests of HMCS should be conservative for inference of positive selection. Indeed, while only 1.95% of the bootstrap values of L_I from the HMCS simulations are below the 2.5% quantile of null distribution, 3.02% of bootstrap values are above the 97.5% quantile. This indicates that while McDonald-Kreitman

style tests of HMCS data are conservative with respect to positive selection, some signatures of negative selection may be spuriously introduced.

In the above analysis, simulations that had either zero polymorphisms or zero fixed differences between human and chimpanzee were excluded from the calculation of L_I, as the statistic is undefined for such values. However, the proportion of simulations that were excluded from the HMCS dataset is remarkably similar to the proportion of simulations that were excluded from the unfiltered dataset (Table S15), suggesting that no bias has been introduced.

References

1. Hudson RR (2002) Generating samples under a Wright-Fisher neutral model of genetic variation. Bioinformatics 18: 337-338.

2. Hwang DG, Green P (2004) Bayesian Markov chain Monte Carlo sequence analysis reveals varying neutral substitution patterns in mammalian evolution. Proc Natl Acad Sci U S A 101: 13994-14001.
